# Dependence of the Fe(II)-Gallic Acid Coordination Compound Formation Constant on the pH

**DOI:** 10.3390/foods10112689

**Published:** 2021-11-03

**Authors:** Franjo Frešer, Gregor Hostnik, Jelena Tošović, Urban Bren

**Affiliations:** 1 Faculty of Chemistry and Chemical Technology, University of Maribor, Smetanova 17, SI-2000 Maribor, Slovenia; franjo.freser@student.um.si (F.F.); gregor.hostnik@um.si (G.H.); jelena.tosovic@um.si (J.T.); 2 Faculty of Mathematics, Natural Sciences and Information Technologies, University of Primorska, SI-6000 Koper, Slovenia

**Keywords:** UV/Vis spectra, gallic acid, Fe(II) ions, coordination compounds, modeling, Job diagrams, density functional theory calculations

## Abstract

One important property of tannins involves their ability to form coordination compounds with metal ions, which is vital for the bioavailability of these ions, as well as for the antibacterial and antioxidative activities of tannins. In this study, the pH dependence of interactions between gallic acid, one of the basic building blocks of tannins, and Fe(II) ions, was investigated using UV/Vis spectroscopy, in conjunction with density functional theory (DFT) calculations. Moreover, two models were developed to explain the processes taking place in the solution. The first model treated the reaction as a simple bimolecular process while the second also considered the protolytic equilibrium, which was proven very successful in discerning the pH dependence of formation constants, and whose assumptions were well supported by DFT calculations. We showed that the two-time deprotonated gallic acid species forms the coordination compound with Fe(II) ions in a 1:1 molar ratio. To gain better insight into the process, the coordination compound formation was also studied using various DFT functionals, which further supported the model results. Furthermore, due to the relatively low sample amounts needed, the methodology developed here will be useful to study compounds that are more difficult to isolate.

## 1. Introduction

**Figure 1:**
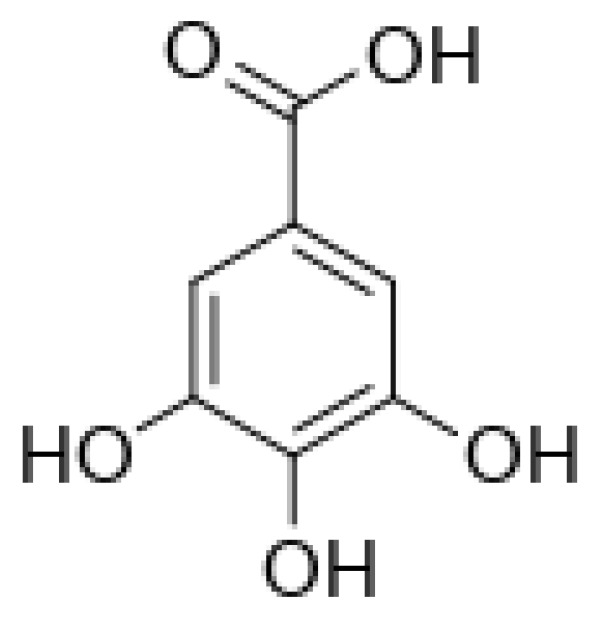
Chemical structure of gallic acid.

Gallic acid (3,4,5-trihydroxybenzoic acid; see Figure 1) represents a compound belonging to the class of polyphenols, and is one of the main products of gallotannin hydrolysis [1]. It can be found in different food sources, e.g., in honey, various berries, pomegranates, and other fruits [2]; vegetables, such as rhubarb [3]; certain beverages, such as green tea [2]; and in nuts, such as pecans [3]. In these sources, gallic acid resides as a free compound or as a building block of larger molecules. Industrially, gallic acid is most often obtained by the enzymatic degradation of tannic acid [1].

The production of iron gall ink likely represents the most widespread application of gallic acid in history [4,5]. Its characteristic blue color is due to the formation of coordination compounds, between gallic acid and iron ions [5,6]. On the other hand, gallic acid has been subjected to several studies, due to its promising antibacterial [7,8,9,10], anti-carcinogenic [11,12,13], and antioxidative [14,15,16] properties. In addition to many beneficial effects on human health, it is also used in spectrophotometry to determine concentrations of certain metal ions, such as calcium, zinc, magnesium, etc. [17], because gallic acid (and its azo derivatives) forms coordination compounds with several metal ions, e.g., Zn(II), Cd(II), Cu(II), Ni(II), Mn(II), Co(II), and Fe(II) [18,19].

The interactions of gallic acid with iron ions are important for several reasons. One vital property of tannins (and gallic acid) involves their antibacterial activity, which is typically ascribed to three mechanisms: (i) interaction of tannins with bacterial and substrate proteins [20]; (ii) interaction with bacterial cell wall plasma membrane [21]; and (iii) chelation of essential metal ions [20,22,23,24]. In the last mechanism of antibacterial action, the interactions of gallic acid with iron ions play a crucial role. Through the formation of coordination compounds with iron ions, the latter are depleted, which prevents the growth of many bacteria, as iron is associated with several key processes in bacterial cells [24].

Tannins (and their building blocks) are also important for their antioxidant potential [3,25]. By binding iron ions, they can prevent (or reduce the intensity of) Fenton-like reactions in a cell and, thus, reduce oxidative stress [26,27]. Most coordination compounds of iron involve ions that are in oxidation state II or III [18], which primarily prefer octahedral structures, so up to three bidentate ligands can bind a single iron ion. The stoichiometry of the coordination compounds of polyphenols with iron ions depends on the pH [28]. Under moderately acidic conditions (pH between 5.00 and 6.50), each iron ion can generally bind two or three polyphenolic ligands [29]. On the other hand, under very acidic conditions (pH less than 4.00), every iron ion binds a single polyphenolic ligand [30].

Another important issue is the effect of the presence of polyphenols on Fe(II)/Fe(III) redox equilibrium. In several previous studies, it was claimed that the presence of polyphenols may prolong the oxidation of Fe(II) into Fe(III) for several days [31]. However, one also finds multiple studies that show that iron(II) ions can oxidize relatively rapidly after the formation of coordination compounds with polyphenols in the presence of oxygen from the solution [32,33]. This can be (at least partially) ascribed to fact that coordination compounds between polyphenolic ligands and Fe(III) ions are generally more stable than coordination compounds of the same ligands with Fe(II) ions [32,33]. On the other hand, after binding of ligands to Fe(III) ions, polyphenolic compounds can also reduce Fe(III) to Fe(II) ions, while oxidizing themselves to semiquinone or quinone molecules [34,35]. Alternatively, in some studies, it was assumed that the presence of polyphenols does not affect redox equilibrium of iron ions at all [36].

To summarize, the knowledge of interactions of tannins with Fe(II) ions is of utmost importance, since it is crucial toward understanding the antibacterial activities [20,22,23,24] and the antioxidative potential of tannins [26,27]. Moreover, the pH dependence of interactions of tannins with Fe(II) ions is not of pure theoretical interest. Tannins represent important food constituents, and the understanding of their interactions with metal ions can help to explain how they influence the bioavailability of essential metal ions in different parts of the digestive tract (the pH in the digestive tract varies from very acidic in the stomach to basic in the intestines). Pure tannins are quite difficult to obtain; on the contrary, gallic acid is easily commercially accessible, making it an excellent model compound. Furthermore, gallic acid was, for centuries, used in the preparation of iron-gal ink [5,6], and the understanding of interactions of gallic acid with Fe(II) ions may be useful in planning the restoration work on antique books.

Although gallic acid is easily accessible, relatively few studies examining the chelation reaction of Fe(II) and Fe(III) ions with gallic acid have been carried out to date [5,6,26,35,36,37]. In these studies, either the thermodynamics, (i.e., stoichiometry and equilibrium constants) [26], or kinetics [35,36] of gallic acid reactions with iron ions were followed. They also differ in the pH range, where the formation of coordination compounds was observed [35,36,37]. Based on previous studies, it might be concluded that every Fe(II) ion binds a single gallic acid in the pH range used in our study [36]. Moreover, the equilibrium constant for Fe(II) ion binding to gallic acid (log(K)≈7) [37] is significantly smaller than the equilibrium constant for Fe(III) ion binding (log(K)≈34) [26], while deprotonation plays an important role in the coordination compound formation [36,37].

The goal of our present study was to obtain better insight into processes taking place during the formation of coordination compounds between gallic acid and Fe(II) ions by fitting model functions to experimental data. The Job method [38], in conjunction with UV/Vis spectroscopy, was used to follow the concentration of coordination compounds in the solution. The obtained experimental data were fitted to a model function in order to obtain the formation constants and to determine which gallic acid species primarily reacts with Fe(II) ions. The proposed reaction mechanism was further confirmed/investigated computationally using the density functional theory.

## 2. Materials and Methods

### 2.1. Experimental Methods

An aqueous solution of gallic acid or FeSO${}_{4}$·7H${}_{2}$O with a concentration of $2\cdot 10^{-2}\;\mathrm{mol}$·$L^{-1}$ was prepared by dissolving gallic acid monohydrate (Sigma-Aldrich, Steinheim, Germany) and FeSO${}_{4}\cdot 7\;$H${}_{2}$O (Emsure, Darmstadt, Germany) in water, respectively. These solutions were further diluted in order to obtain a solution of final concentrations for both gallic acid and iron(II) ions. Stock solutions were prepared freshly every day. Solutions of present concentrations were prepared by diluting the stock solutions. The desired pH of the studied solutions was maintained using 50 mM acetate buffer prepared from glacial acetic acid (Emsure, Darmstadt, Germany), sodium acetate (Emsure, Darmstadt, Germany), and type I water.

All experimental spectra measurements were performed using a Varian Cary 50 UV-Vis spectrophotometer (Varian Inc., Mulgrave, Australia), equipped with a single cuvette thermostat holder Single Cell Peltier Accessory (Varian Inc., Mulgrave, Australia) at a temperature of 298.15 K in the wavelength range from 800 to 200 nm. All measurements were performed in the quartz cuvettes. The measurement step was 1 nm and the average time for each step was 0.2 s. A Metrohm 780 pH Meter (Metrohm, Herisau, Switzerland) equipped with a BioTrode combined electrode (Metrohm, Herisau, Switzerland) was applied to monitor the pH directly in the cuvette.

First, the spectra of gallic acid and gallic acid bound to the iron ions were measured. The spectra were recorded once every hour. This was repeated until two identical consecutive spectra were obtained in order to confirm that the system had reached its equilibrium.

To construct the Job plot, five sets of samples were prepared in acetate buffer ($c_{\mathrm{acetate}\;\mathrm{buffer}}$ = 50 mM) at five different pH values (pH = 3.52–5.50). Each set contained 21 samples. The concentration of gallic acid and Fe(II) ions continuously varied in a way that the sum of concentrations of gallic acid and iron(II) ions was kept constant ($c=5\cdot 10^{-4}\;\mathrm{mol}\cdot L^{-1})$. Three measurements were performed for each individual sample in the set (λ=560nm, T=298.15K and the average time for each measurement was 5 s).

### 2.2. Computational Methods

All calculations were performed using the Gaussian 16 program package [39]. All compounds were initially geometry-optimized at the B3LYP level of theory [40,41]. Singlet and quintet states were applied for complexes of Fe(II), whereas doublet and sextet states were applied for complexes of Fe(III) [40,42]. The LANL2DZ effective core potential basis was used to describe the metal center, whereas the 6-311+G(d,p) basis set was employed for all remaining atoms [41,43]. All species were optimized in implicit solvent (water, dielectric constant = 78.3553) using the conductor-like polarizable continuum model (CPCM) [44]. The allocation of equilibrium geometries was confirmed by analyzing the results of the subsequent vibrational frequency calculations in the harmonic approximation: only real frequencies were obtained. To confirm which of the investigated coordination complexes is the most stable for each Fe(II) and Fe(III) ion, the geometries in the most stable spin state were additionally fully re-optimized using M06 and ωB97XD functionals [45,46].

The standard reaction Gibbs free energy ($\Delta G_{f}^{⦵}$) for forming a complex from its infinitely separated ligands and solvated central ion was calculated using B3LYP, M06, and ωB97XD functionals, following Equation (1):

(1) $$\Delta G_{f}^{⦵}=\sum_{products}G_{f}^{⦵}-\sum_{reactants}G_{f}^{⦵}$$

The $\Delta G_{f}^{⦵}$ of the investigated reactions were determined at *T* = 298.15 K and *P* = 101,325 Pa. To simulate the UV/Vis spectra of the most stable gallic acid coordination compounds of Fe(II) and Fe(III) ions in an aqueous solution, the time-dependent density functional theory (TDDFT) was utilized. For this purpose, B3LYP, M06, ωB97XD, and CAM-B3LYP functionals were employed [43,47,48,49]. Because the B3LYP and M06 methods overestimate all wavelength values, their results were scaled using scaling factors of 0.82 and 0.78, respectively. Molecular orbitals (MOs) were generated using Chemcraft graphical software for visualization of quantum chemistry computations (https://www.chemcraftprog.com; version 1.8, build 552b). Cartesian coordinates and associated energies are presented in the Supplementary Materials.

### 2.3. Data Treatment

Two different models (model A and model B) were set up to describe the reaction between gallic acid and iron(II) ions. Model parameters (equilibrium constants $K_{\mathrm{eq}}$ and molar absorption coefficient ϵ) were obtained by fitting a model function to the experimental data using the least squares procedure. The reported uncertainties were calculated using the bootstrapping method. Experimental points were randomly shifted in the interval from 0.95 to 1.05 of the measured values and newly fitted with the model function. This procedure was repeated 100 times, and the error of the fitted parameters is given as the standard deviation of the model parameters.

#### 2.3.1. Model A—Bimolecular Reaction

Initially, a simple bimolecular reaction between gallic acid (GA) and Fe(II) in a 1:1 ratio was assumed, as presented in Equation (2). The last step in the equation represents the oxidation of Fe(II) into Fe(III), which takes place after binding of Fe(II) onto gallic acid. A blue colored solution arises from the gallic acid coordination compound with Fe(III) [5,6]. The formation of the gallic acid coordination compound with the Fe(III) ion is assumed to be irreversible: the Fe(III) ion bound to gallic acid cannot be exchanged with Fe(II) ion anymore due to the much larger formation constant with Fe(III), compared with the Fe(II) [28,37].

(2) $$\mathrm{GA}+\mathrm{Fe}(\mathrm{II})\overset{K_{\mathrm{eq},A}}{⇌}{\left[\mathrm{GAFe}(\mathrm{II})\right]}^{2+}\overset{\mathrm{oxidation}}{⟶}{\left[\mathrm{GAFe}(\mathrm{III})\right]}^{3+}$$

After derivation (see Supplementary Materials for details), the polynomial was obtained for the equilibrium concentration of the coordination compound formed ([[GAFe(II)]${}^{2+}$]). The model value of absorbance (Equation (3)) was then expressed by using Beer–Lambert’s law:

(3) $$A_{\mathrm{model}\;A}=[{\left[\mathrm{GAFe}(\mathrm{II})\right]}^{2+}]\cdot \epsilon \cdot b=\frac{1+K_{\mathrm{eq},A}\cdot c-\sqrt{1+2\cdot K_{\mathrm{eq},A}\cdot c+{\left[K_{\mathrm{eq},A}\cdot c\cdot \left(1-2\cdot x_{\mathrm{GA}}\right)\right]}^{2}}}{2\cdot K_{\mathrm{eq},A}}\cdot \epsilon \cdot b$$

#### 2.3.2. Model B—Effect of the Protolytic Equilibrium

**Figure 2:**
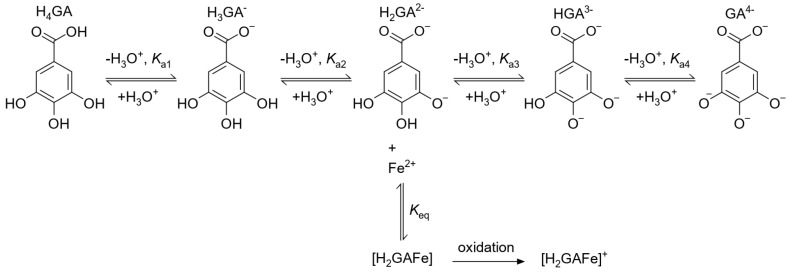
Model B reaction scheme in which $H_{2}{\mathrm{GA}}^{2-}$ reacts with iron(II) ions. This forms a coordination compound ${[H}_{2}\mathrm{GAFe}]$, which later irreversibly oxidizes into [H${}_{2}$GAFe]${}^{+}$.

Model B was developed in order to explain the pH dependence of the formation constant, $K_{\mathrm{eq},A}$, determined by Model A. In Model B, it is assumed that different species of gallic acid are in a protolytic equilibrium [50], and that only one of these species ($H_{2}{\mathrm{GA}}^{2-}$) reacts with Fe(II) ions, as depicted in the reaction scheme shown in Figure 2. The final, irreversible oxidation of Fe(II) to Fe(III) in the coordination compound was treated in an analogous way as in the case of Model A. Model functions for reactions where alternative protonation species of gallic acid react individually with Fe(II) ions were also derived and fitted to the experimental data (see Supplementary Materials for details).

The total gallic acid concentration can be written as a sum of the concentrations of individual species (see Supplementary Materials Equation (S6)). The mass balance for Fe(II) ions in Model B can be written analogously as in the case of model A. For an easier comparison of the results obtained by Models A and B, an apparent equilibrium constant *U* (Equation (4)) was introduced.

(4) $$U_{{[H}_{2}\mathrm{GAFe}]}=\frac{K_{\mathrm{eq},B}\cdot K_{a1}\cdot K_{a2}}{z\cdot {[H}_{3}O^{+}{]}^{2}}$$

The model function B (Equation (5)) was obtained by combining the physically meaningful solution of the quadratic equation with the negative sign before the square root (see Supplementary Materials for details) and by applying Beer–Lambert’s law:

(5) $$A_{\mathrm{model}\;B}={[[H}_{2}\mathrm{GAFe}]]\cdot \epsilon \cdot b=\frac{1+U\cdot c-\sqrt{1+2\cdot U\cdot c+\left[U\cdot c\cdot (1-2\cdot x_{\mathrm{GA}}\right){]}^{2}}}{2\cdot U}\cdot \epsilon \cdot b$$

## 3. Results and Discussion

The formation of gallic acid Fe(II) ion coordination compounds results in a large change in the UV/Vis spectrum. As demonstrated by Lu et al. [36], after the formation of coordination compounds, a new, wide peak appears with its maximum at wavelengths between 560 and 570 nm, while both peaks of gallic acid are red shifted. In the experimental spectrum obtained in this study (Supplementary Materials, Figure S2), the redshift is slightly less pronounced than the one observed by Lu et al. This can be at least partly ascribed to the effect of the pH (5.00 in Lu et al. compared to 5.50 in this study), while discrepancies at lower wavelengths are very likely the consequence of buffer absorption. However, the changes in the UV/Vis spectrum due to the coordination compound formation observed in the present study are still qualitatively the same as the ones observed in Lu et al. [36].

### 3.1. Job Plot

**Figure 3:**
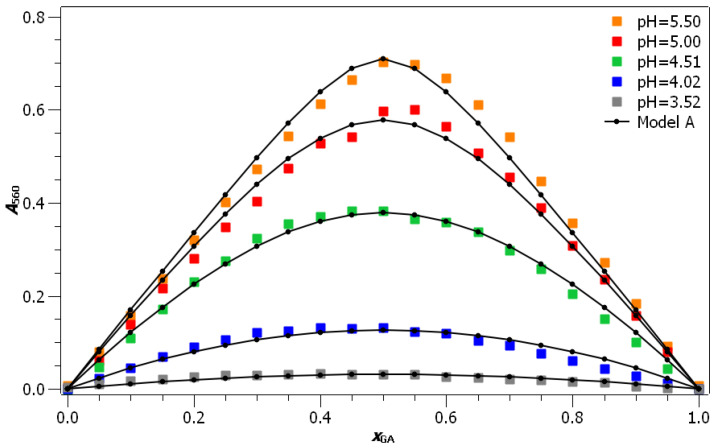
Model function A in comparison to experimental results for sets with $c\;=\;5\cdot 10^{-4}\;\mathrm{mol}\cdot L^{-1}$ ($R^{2}=0.995$).

According to the results of the UV/Vis experiments, the wavelength λ=560nm was chosen for the construction of the Job diagram. In Figure 3, the Job plot at a wavelength of 560 nm and a temperature of 298.15 K is plotted for different pH values. Two main observations can be made from this Job plot: (i) the absorbance values increase with increasing pH of the solutions, indicating that a larger concentration of coordination compounds is formed in solutions with higher pH values. (ii) The maximum absorbance of UV/Vis light can be observed in solutions with equimolar concentrations of gallic acid and Fe(II) ions, indicating that, at the given conditions, gallic acid reacts with Fe(II) ions in a 1:1 ratio. This is further confirmed by the extrapolation of the first and the last six points of the Job plot (see Figures S3 and S4 of Supplementary Materials), where the $x_{\mathrm{GA}}=0.50$ value of intercept corresponds to the gallic acid:Fe(II) ratio of 1:1. The obtained values are reported in Table 1. At higher pH values, the determined molar fraction of gallic acid is slightly higher than 0.50 (0.52 and 0.53 at pH values of 5.00 and 5.50, respectively), and at lower pH values it is somewhat lower than 0.50 (0.42 and 0.48 at pH values of 4.02 and 4.51, respectively). The increase in this ratio with the increasing pH could be due to changes in the stoichiometry of the coordination compound. If this were the case, raising the pH would result in more coordination compounds with a stoichiometry of 1:2 or 1:3 being formed (as proposed by Perron and Brumaghim [28]). On the other hand, it can also e safely assumed that the differences in values are due to measurement and extrapolation errors. The second case seems more likely because the extrapolation error at low pH values (where significant deviation from the value of 0.5 is observed) is significantly higher than at high pH values due to the flattening of the curves in the Job plot with decreasing pH values (see Figures S3 and S4 of Supplementary Materials). For pH = 3.52, no extrapolation was performed, because the values are burdened with a large experimental error due to the low absorbance and with a flat curve in the Job diagram.

In addition to the absorbance at λ=560nm, the pH of the solutions was also monitored. The measured values are collected in Figures S4 and S5 of the Supplementary Materials. The changes of pH values are in the range of the experimental error or slightly bigger than the experimental error. A practically constant pH is, of course, an expected consequence of buffer solution applications. However, in all cases, a slight decrease in the pH value was observed in the vicinity of the ratio $x_{\mathrm{GA}}=0.5$ (the pH = 3.50 again represents a slight exception). This indicates that during the coordination compound, formation oxonium ions are released, as was already proposed by several investigations [36,37]. The decrease in the pH value is slightly more pronounced at higher pH values. This is very likely due to two reasons: (i) a larger amount of coordination compounds is formed at higher pH values; and (ii) because acetic acid p$K_{a}$ value is 4.76 [51], the buffer capacity at the pH of 5.50 is somewhat smaller than at pH values closer to 4.76.

In all, the main conclusions that may be drawn directly from the Job plots are: (i) in the studied pH range gallic acid and Fe(II) ions react in a 1:1 ratio; (ii) a larger amount of coordination compounds is formed at higher pH values; and (iii) pH measurements (see Figures S4 and S5 of Supplementary Materials) indicate that oxonium ions are released during the coordination compound formation. In order to obtain the formation constant of the coordination compound between gallic acid and Fe(II) ions, as well as to explain its dependence on the pH, two different models were fitted to the experimental data.

### 3.2. Model A—Simple Bimolecular Reaction

Based on the data derived from the Job plot, Model A was developed (see Equation (2)), in which the formation of the coordination compound was presented as a simple bimolecular reaction between gallic acid and Fe(II) ions. Equation (3), derived under this assumption, was fitted to the experimental data. During the fitting procedure, the ϵ was kept constant for all pH values, while $K_{\mathrm{eq},A}$ formed an adaptable parameter for each pH. The comparison between experimental and model values of absorbance for sets with c=5·$10^{-4}\;\mathrm{mol}$·$L^{-1}$ is depicted in Figure 3. Because equilibrium absorbance at pH 3.52 was rather low, two additional sets at low pH values (pH = 3.52 and pH = 4.24) and higher concentrations were measured, and also fitted to Model A (see Supplementary Materials, Figure S7 for the depiction of the comparison between experimental and model values). The agreement between Model A and the experimentally determined values is excellent for all pH values, except for pH = 3.52. The reason for the deviation can be, at least for the Job plot at $c=5\cdot 10^{-4}\;\mathrm{mol}\cdot L^{-1}$, ascribed to low values of absorbance, as well as to low $K_{\mathrm{eq},A}$ values, which result in a relatively wide Job plot. The agreement between experimental and calculated values at higher concentrations (see Figure S7) was excellent for the pH value of 4.24, while due to the extremely wide peak in the Job diagram, this agreement was worse at pH = 3.52.

**Figure 4:**
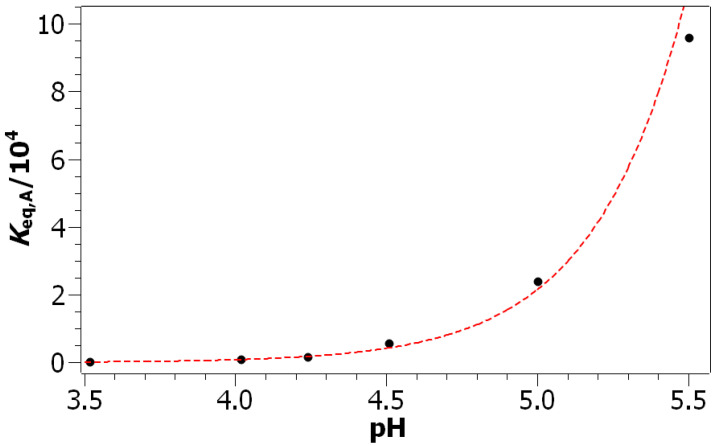
Dependence of the formation constant of the coordination compound [GAFe(II)]${}^{2+}$ on the pH of the acetate buffer.

In Table 2, the $\epsilon_{\mathrm{model}\;A}$ and $K_{\mathrm{eq},A}$ parameter values obtained by fitting Model A to the experimental values are presented. The value of the molar absorption coefficient ϵ determined with Model A is very close to its average value determined from the extrapolation of the start and end points (${\bar{\epsilon }}_{\mathrm{extrapolated}}=3369.3\;L$·${\mathrm{mol}}^{-1}$·${\mathrm{cm}}^{-1}$, $\epsilon_{\mathrm{model}\;A}=3476.0\;L$·${\mathrm{mol}}^{-1}$·${\mathrm{cm}}^{-1}$). The second result of fitting Model A to experimental values are the formation constants. Their values increase with the increasing pH and are depicted as a function of the pH in Figure 4. It can be seen that the formation constants are increasing exponentially with the increasing pH. To explain this dependence of formation constants on the pH, a new model (Model B) had to be introduced.

### 3.3. Model B—Effect of the Protolytic Equilibrium

Taking into account the fact that the equilibrium constant is increasing exponentially with the increasing pH value, and the small decrease in pH values of the studied solutions that correlates with the increase in the $A_{560}$ value, a new model had to be devised to obtain the explanation for the pH-dependence of the formation constant $K_{\mathrm{eq},A}$. Model B was therefore developed by introducing the gallic acid protolytic equilibrium.

**Figure 5:**
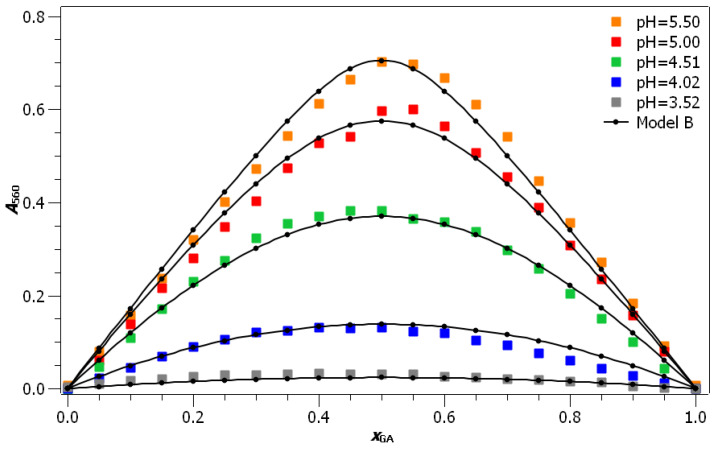
Model function B fitting to the experimental results ($c=5\cdot 10^{-4}\;\mathrm{mol}\cdot L^{-1})$, where it is assumed that $H_{2}{\mathrm{GA}}^{2-}$ protonation species reacts with Fe(II) ions to form the coordination compound ${[H}_{2}\mathrm{GAFe}]$ ($R^{2}=0.994$).

**Figure 6:**
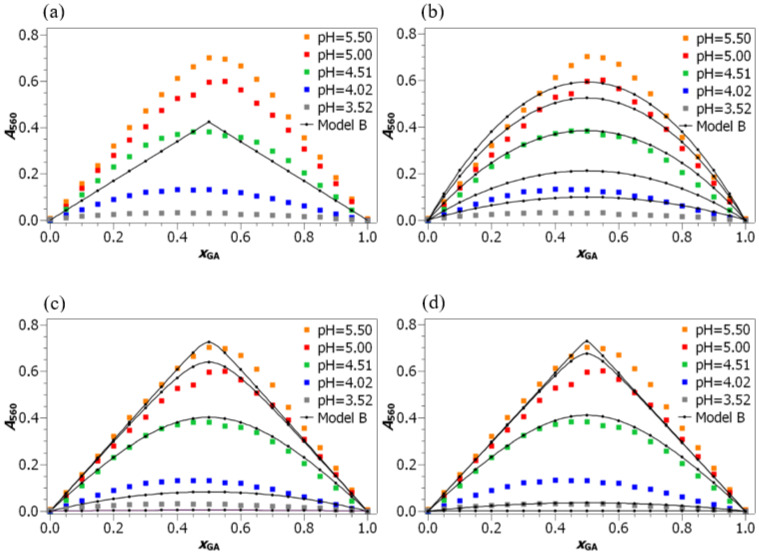
Different model functions B fitting with the results of experiments ($c\;=\;5\cdot 10^{-4}\;\mathrm{mol}\cdot L^{-1})$. We assumed that different types of gallic acid react with iron(II), which leads to the formation of the following coordination compounds: (**a**) ${[H}_{4}{\mathrm{GAFe}]}^{2+}$, (**b**) ${[H}_{3}{\mathrm{GAFe}]}^{+}$, (**c**) ${\left[\mathrm{HGAFe}\right]}^{-}$, (**d**) ${\left[\mathrm{GAFe}\right]}^{2-}$.

The comparison between the model function B (Equation (5)) and the experimental data for sets with $c=5\cdot 10^{-4}\;\mathrm{mol}\cdot L^{-1}$ is depicted in Figure 5 and Figure 6. For the agreement between the experimental data and Model B for additional sets at higher concentrations, seeSupplementary Materials Figure S9. The agreement between Model B and the experimental data were tested for all protonation species of gallic acid possibly present in the solution. It can be seen that when species H${}_{4}$GA (Figure 6a) was chosen as the reactive species, the model totally failed to correctly describe the experimental data. This is hardly surprising, because the concentration of H${}_{4}$GA species decreases with the increasing pH, while the concentration of the coordination compound increases.

The agreement between the experimental and model values is better in the case of the remaining protonation species. For one deprotonated species, $H_{3}{\mathrm{GA}}^{-}$ (See Figure 6b), the model correctly predicts an increase in $A_{560}$ with the increasing pH. However, this increase is not as pronounced as it should be according to the experimental data. This can be explained with the relatively high concentrations of the H${}_{3}$GA${}^{-}$ species already at the lowest studied pH values. This is the reason why its concentration cannot significantly increase, (i.e., by several orders of magnitude). Moreover, the predicted Job curves are simply too wide. This is again the consequence of the relatively high H${}_{3}$GA${}^{-}$ species concentrations and of the corresponding low $K_{\mathrm{eq},B}$ value.

In contrast, the reason for the disagreement between the experimental and model data in the case of HGA${}^{3-}$ and GA${}^{4-}$ species (depicted in Figure 6c,d, respectively) can be attributed to the extremely low concentrations of these species in the studied pH range. This results in high values of formation constants $K_{\mathrm{eq},B}$, which leads to very steep curves in the Job plot. Moreover, the model values of $A_{560}$ for these two species also increase faster with the increasing pH than the experimental $A_{560}$ values.

**Figure 7:**
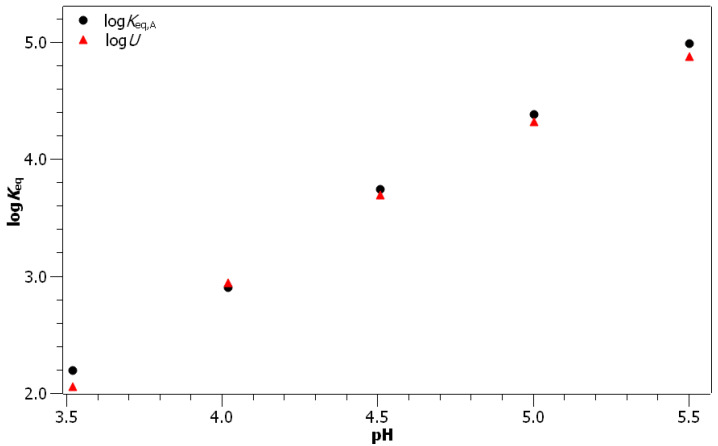
Dependence of log($K_{\mathrm{eq},A}$) and log($U_{{[H}_{2}\mathrm{GAFe}]}$) values on the pH. The only significant difference between the values occurs at pH 5.50, where, however, the experimental error of $K_{\mathrm{eq},A}$ is quite big. See Figure S13 for the depiction with the estimated fitting errors.

Finally, an excellent agreement between the experimental and model values was obtained when H${}_{2}$GA${}^{2-}$ was chosen as the reactive species (Figure 5). This agreement is as good as in the case of Model A. This is even more exceptional if one considers that there were six free parameters used in the fitting of Model A, while there were only two in the case of Model B. For comparison of the results obtained using Models A and B, the formation constants ($K_{\mathrm{eq},A}$ calculated with Model A and $U_{{[H}_{2}\mathrm{GAFe}]}$, which is the $K_{\mathrm{eq},A}$ equivalent in Model B) should be compared. To that end, the values of log($K_{\mathrm{eq},A}$) and log($U_{{[H}_{2}\mathrm{GAFe}]}$) are depicted in Figure 7. It can be seen that the values determined using Model B are in a very good agreement with the values obtained using Model A. The only significant difference between the values $K_{\mathrm{eq},A}$ and $U_{{[H}_{2}\mathrm{GAFe}]}$ can be observed at the pH of 5.50, where the experimental error in $K_{\mathrm{eq},A}$ is rather big.

Because Model B, for the reaction of the twice deprotonated gallic acid H${}_{2}$GA${}^{2-}$ with Fe(II) ions is in excellent agreement with the experimental data, while the reactions of the remaining gallic acid protonation yield, at best, a very limited agreement with the experimental data, it may be safely concluded that the H${}_{2}$GA${}^{2-}$ species is the one that reacts with Fe(II) ions in the process of coordination compound formation in the pH range studied. This conclusion agrees well with the ability of polyphenols to form coordination compounds with a five-member ring structure, with two aromatic carbons, two phenolic oxygen atoms, and a divalent metal ion in its corners [28].

The formation constant for Model B is together with the extinction coefficient ϵ reported in Table 3. The formation constant $K_{\mathrm{eq},B}$ can be compared to the one obtained using potentiometric titration. Its value, determined by Powell and Taylor [37] ($1\cdot 10^{7}$), is of the same order of magnitude as the one obtained in this study. However, there are two important differences between the results obtained in this study and the results obtained in the study of Powell and Taylor [37]: (i) in the study of Powell and Taylor, the coordination compound formation was observed at a higher pH value, which is a direct consequence of two distinct investigation methods used. (ii) Three-time deprotonated gallic acid HGA${}^{3-}$ was assumed to be the reactive species according to the results of their study [37].

### 3.4. Fractions of Species

The use of the model function B with $H_{2}{\mathrm{GA}}^{2-}$ as the reactive protonation species facilitates the calculation of the equilibrium concentrations (and molar fractions) of individual species in the solutions of various compositions.

**Figure 8:**
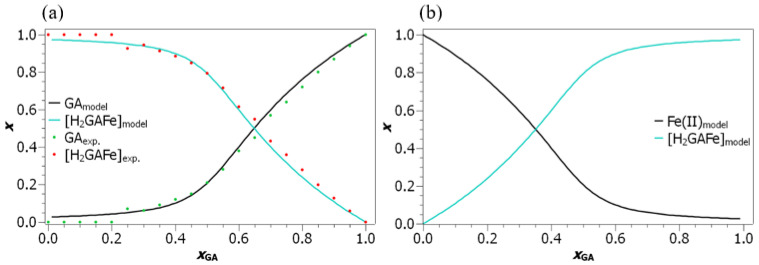
(**a**) Dependence of the molar fraction (*x*) of gallic acid present in the free form and of gallic acid bound in the coordination compound on $x_{\mathrm{GA}}$ for sample sets with the pH of 5.50. (**b**) Dependence of the molar fraction (*x*) of iron(II) ions present in the free form and iron(II) ions bound in the coordination compound on $x_{\mathrm{GA}}$ for sample sets with the pH of 5.50.

In Figure 8 the model values of molar fractions of gallic acid bound in the coordination compound, as well as of gallic acid in the free form at the pH of 5.50, are compared to the experimentally determined values. In order to experimentally determine the molar fraction of the free and bound gallic acid, the UV/Vis spectrum was measured for every solution composition. The spectrum was then reproduced as a linear combination of the spectrum of free gallic acid and on the spectrum of the coordination compound. The percentage of the spectrum of gallic acid and the coordination compound required to reproduce the experimental solution spectrum was then interpreted as the molar fraction of the free and bound gallic acid, respectively. The model values obtained at the remaining pH values are presented in Supplementary Materials Figure S10, while their experimental values for the molar fraction of the free and bound gallic acid were not accessible. This is due to the fact that the spectrum of the coordination compound could not be measured at lower pH values, because of the extremely high excess of Fe(II) ions required to measure the coordination compound spectrum.

When experimentally obtained molar fractions are compared to the model values, we may see that the agreement is extremely good, which gives an additional confirmation of the chosen model. Moreover, this facilitates the estimation of the gallic acid species present in the solution. Not surprisingly, when iron ions are in excess (at low $x_{\mathrm{GA}}$ values), practically all gallic acid is bound into the coordination compound, while with increasing $x_{\mathrm{GA}}$ values, the molar fraction of gallic acid bound into the coordination compound is, as anticipated, decreasing. Of course these trends are just the reverse for iron ions, as can be seen from Figure 8b. If these molar fractions are compared to their values at the remaining pH values (see Figures S9 and S10 of the Supplementary Materials), one may deduce that the molar fraction of gallic acid and iron ions in the coordination compound is decreasing with the decreasing pH. Namely, at equimolar solution compositions, roughly 79.4% of gallic acid is bound into the coordination compound at the pH of 5.50, while at the pH of 3.52, practically all gallic acid (97.3%) is free in the solution. An analogous pH dependence can, according to the model values, be observed for the iron ions.

### 3.5. Computational Considerations

To confirm the proposed Model B reaction scheme (Figure 2), which predicts that Fe(II) ions preferentially react with H${}_{2}$GA${}^{2-}$ gallic acid protonation species to form the [H${}_{2}$GAFe] coordination compound, which subsequently oxidizes into [H${}_{2}$GAFe]${}^{+}$, additional computational investigations were performed.

**Figure 9:**
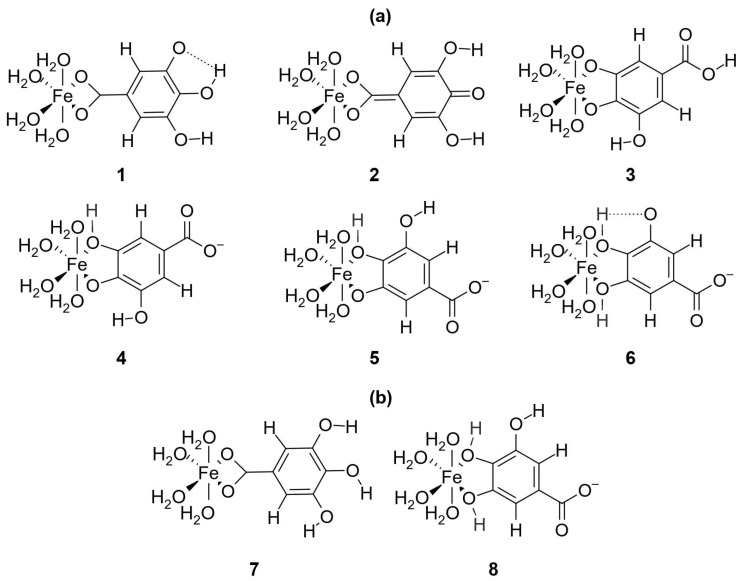
Chemical structures of all proposed coordination complexes between iron ions and gallic acid anion (**a**) or monoanion (**b**), which were considered in the DFT calculations. The numbering below the coordination compounds is used in the main text to refer to these complexes.

**Figure 10:**
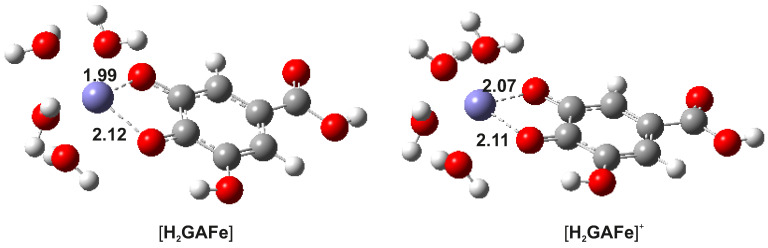
Optimized geometries of the most stable octahedral 1:1 complexes of Fe(II) (left) and Fe(III) (right) ions with H${}_{2}$GA${}^{2-}$ gallic acid protonation species (bond distances are given in Å).

The first task was to obtain the most favorable structure of the Fe(II) and Fe(III) coordination compound. Considering that our experimental results show that iron ions and gallic acid react in a 1:1 stoichiometric ratio, six octahedral coordination complexes of this type for both Fe(II) and Fe(III) with H${}_{2}$GA${}^{2-}$ were proposed and further examined (Figure 9). High-spin states (quintet multiplicity for Fe(II) and sextet multiplicity for Fe(III)) were compared against low-spin states (singlet multiplicity for Fe(II) and doublet multiplicity for Fe(III)). In all cases, the high-spin state complexes are significantly more stable (by ∼30–35 kcal · mol${}^{-1}$ in the majority of cases) than the same complexes in the low-spin state, indicating that the H${}_{2}$GA${}^{2-}$ represents a weak-field ligand (see Table S2 of the Supplementary Materials). Moreover, among all proposed structures in the high-spin state, all three density functional theory (DFT) methods employed (B3LYP, M06, and ωB97XD) predict that complex 3 represents the most stable coordination compound for both Fe(II) and Fe(III) ions (Figure 10 and Table S2). Complex 3 is more stable than complex 2 by ∼ 5–6 kcal · mol${}^{-1}$. All remaining complexes are generally more than 10 kcal · mol${}^{-1}$ less stable than complex 3. In addition, complex 6 represents the least stable complex. The optimized geometries of the most stable complex 3 of Fe(II) and Fe(III) ions, with relevant bond distances indicated, are depicted in Figure 10. These two structures were subjected to subsequent analyses. The observation that the iron ions preferentially coordinate to phenolic oxygen atoms is in accordance with several experimental studies [18,28].

**Figure 11:**
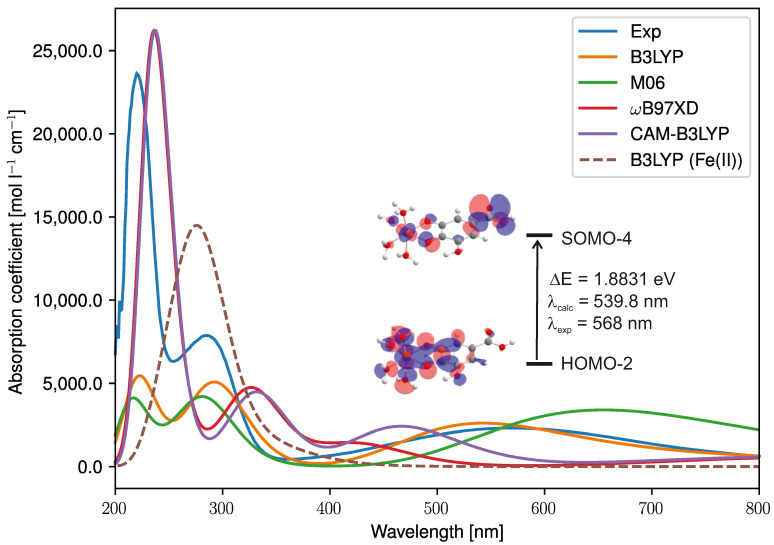
Comparison of the experimental (blue line) and the simulated spectra of [H${}_{2}$GAFe]${}^{+}$ (full line) and [H${}_{2}$GAFe] (dashed line). DFT functionals were used in combination with LANL2DZ and 6-311+G(d,p) basis sets for Fe and the remaining atoms, respectively, as well as with the CPCM solvation model. The orbitals involved in the transition responsible for the broadest band at high wavelengths are depicted (HOMO, SOMO, and LUMO denote the highest-, singly, and lowest-(un)occupied molecular orbitals, respectively). In addition, the excitation energy and wavelength (experimental and calculated) of this transition are provided.

The UV/Vis spectra of [H${}_{2}$GAFe] and [H${}_{2}$GAFe]${}^{+}$ complexes were simulated using TDDFT and compared to the experimental spectrum (Figure 11). In general, the simulated spectrum of the Fe(III) complex qualitatively describes the experimental spectrum, well in the case of all four DFT methods employed (B3LYP, M06, ωB97XD, and CAM-B3LYP; Figure 11, full lines). More precisely, all four functionals predict the presence of the characteristic broad band at higher wavelengths. On the other hand, this band is not observed in the case of the spectrum of the Fe(II) complex, which is characterized by with a single peak at lower wavelengths (Figure 11, dashed line). Moreover, the performance of the four functionals employed should be discussed. Namely, from Figure 11, it can be seen that the best agreement between experimental and calculated spectra of the Fe(III) complex is achieved in the case of the B3LYP functional. The only discrepancy between these two spectra lies in the underestimated intensity of the peak observed at the lowest wavelengths in the case of the calculated spectrum. The M06 functional displays a similar performance to B3LYP, when the intensities and wavelengths of the two peaks at lower wavelengths are considered. However, the broad band at higher wavelengths is overestimated. The ωB97XD and CAM-B3LYP functionals show a similar performance. In both cases, the intensities and the wavelengths of the two peaks at lower wavelengths are well reproduced, whereas the wavelength of the characteristic broad band is underestimated.

To assist in the assignment of the absorption bands, a detailed analysis of the TDDFT results was further conducted. The vertical transition wavelengths ($\lambda_{\max}$), oscillator strengths (*f*), excitation energies (ΔE), and orbital contribution coefficients of all absorption bands calculated using the B3LYP functional are reported in Supplementary Materials Table S3. Only strong electron excitations with significant oscillator strengths (f>0.01) were considered to elucidate the absorption properties. The broad absorption band characterized by the maximum at 568 nm (experimental) originates from the HOMO -1 → SOMO -4 (93.7 %) transition, which represents a metal-to-ligand charge transfer (MLCT) transition in the origin, mixed with an intraligand charge transfer (ILCT) character (Figure 11 and Supplementary Materials Table S3). Three individual electronic transitions are responsible for the second absorption band at 285 nm (experimental): HOMO → SOMO -2 (78.6 %), SOMO → LUMO (58.7 %), and HOMO -4 → SOMO -4 (77.6 %). These three electronic transitions correspond to the ligand-to-metal charge transition (LMCT) mixed with ILCT, ILCT, and MLCT, respectively (Supplementary Materials Figure S15 and Table S3). The highest intensity band (Figure 11) at 220 nm (experimental) may be assigned to a combination of ILCT (SOMO -1 → LUMO +2 (51.8 %)), MLCT (HOMO -7 → SOMO -4 (62.4 %)), and MLCT mixed with ILCT (HOMO -1 → SOMO (83.0 %)) transitions.

To confirm the experimental finding that Fe(II) preferably reacts with H${}_{2}$GA${}^{2-}$ compared to H${}_{3}$GA${}^{-}$, the most stable complex of Fe(II) with H${}_{3}$GA${}^{-}$ had to be determined as well. This was achieved by following an identical procedure, as in the case of Fe(II) complexes with H${}_{2}$GA${}^{2-}$. Two different structures (7 and 8, Figure 9) of the [H${}_{3}$GAFe]${}^{+}$ complex were proposed. As in the case of [H${}_{2}$GAFe] and [H${}_{2}$GAFe]${}^{+}$ complexes, the high-spin state coordination compounds are more stable than the low-spin state ones (Supplementary Materials Table S2). Moreover, complex 7 is ∼20 kcal · mol${}^{-1}$ more stable than complex 8 (Supplementary Materials Table S2).

Thermodynamic calculations were further performed to determine the standard Gibbs formation free energy $\Delta G_{f}^{⦵}$ for complexes of Fe(II) with H${}_{2}$GA${}^{2-}$ and H${}_{3}$GA${}^{-}$, respectively, according to the formation equilibrium shown in Equations (6) and (7):

(6) $$H{}_{2}\mathrm{GA}{}^{2-}+[\mathrm{Fe}(H{}_{2}O){}_{6}]{}^{2+}⇌[H{}_{2}\mathrm{GAFe}(H{}_{2}O){}_{4}]+2H{}_{2}O$$

(7) $$H{}_{3}\mathrm{GA}{}^{-}+[\mathrm{Fe}(H{}_{2}O){}_{6}]{}^{2+}⇌[H{}_{2}\mathrm{GAFe}(H{}_{2}O){}_{4}]{}^{+}+2H{}_{2}O$$

In addition, the standard Gibbs formation free energy $\Delta G_{f}^{⦵}$ of the [H${}_{2}$GAFe]${}^{+}$ complex was calculated according to Equation (8):

(8) $$H{}_{2}\mathrm{GA}{}^{2-}+[\mathrm{Fe}(H{}_{2}O){}_{6}]{}^{3+}⇌[H{}_{2}\mathrm{GAFe}(H{}_{2}O){}_{4}]{}^{+}+2H{}_{2}O$$

The standard Gibbs formation free energies were calculated using B3LYP, M06, and ωB97XD functionals. The obtained results are in excellent mutual agreement and indeed indicate that the formation of the [H${}_{2}$GAFe] complex is preferred over that of [H${}_{3}$GAFe]${}^{+}$, whereas the formation of the [H${}_{2}$GAFe]${}^{+}$ complex represents the thermodynamically most favorable complexation reaction (see Supplementary Materials Table S4). All computational results, therefore, qualitatively support the reaction scheme proposed in Figure 2.

### 3.6. Oxidation of Fe(II) in Fe(III) and Fitting of the Model

An important remaining issue to discuss is the oxidation of Fe(II) to Fe(III) after binding to gallic acid. Fe(II) spontaneously oxidizes to Fe(III) in an aqueous solution in the presence of oxygen. However, in the studied pH range the reaction is relatively slow until Fe(II) becomes bound to the gallic acid, when the reaction of oxidation of Fe(II) into Fe(III) becomes rapid [28]. The reason behind the relatively fast oxidation of Fe(II) into Fe(III) after the formation of the coordination compound is the higher stability of Fe(III) coordination compound compared to Fe(II) coordination compound. After the Fe(II) oxidation, the color of the solution changes into blue [5,6], which facilitates detection of the coordination compound using UV/Vis spectroscopy. Because the formation constant of the coordination compound with Fe(III) ($1\cdot 10^{14}$) [28] is several orders of magnitude higher than the formation constant of the formation coordination compound with Fe(II) ($1\cdot 10^{7}$) [37], it may be safely assumed that, after the Fe(III) ion is bound, it can no longer be displaced from the coordination compound by an Fe(II) ion.

It can be argued that, after a long time, all the Fe(II) would be eventually oxidized into Fe(III) and, therefore, the reaction of Fe(III) and gallic acid would be investigated. As this indeed represents the long-term limit, even in the absence of gallic acid, there are two reasons to assume that this is not the case in the time frame of our experiments: (i) after a short time, the $A_{560}$ reaches a plateau and remains stagnant for several hours. (ii) The coordination compound precipitates in the case of the experiment performed with Fe(III) ions under the conditions used in our experiments. The precipitation was not observed in the tested solutions even after an extended period of time (16 h), confirming the assumption that only the Fe(II) bound to gallic acid was oxidized into Fe(III), and then remained bound onto the gallic acid for the duration of the experiment. Taking everything into consideration, it can be safely assumed that no significant error is introduced into the modeling by treating the last reaction step of Fe(II) oxidation as a process that enables the detection of the formed coordination compound, while it does not significantly affect the determined model parameters. For example, the formation constant we determined is of the same order of magnitude as the one determined by Powell and Taylor [37].

## 4. Conclusions

The knowledge of the mechanism of how pH affects the interactions of tannins with metal ions is not of pure theoretical interest. Tannins represent important food constituents, and the understanding of their interactions with metal ions can help explain how they influence the bioavailability of essential metal ions in different parts of the digestive tract (the pH in the digestive tract varies from very acidic in the stomach to basic in the intestines). Moreover, recently, tannins have become popular as antimicrobial feed additives, and one of their possible modes of antibacterial action involves the depletion of essential metal ions from the microorganisms. The knowledge of the pH dependence of tannins’ ability to form coordination compounds is, thus, of crucial importance in understanding their antibacterial effectiveness.

Overall, by using the method of continuous variation in the pH range between 3.52 and 5.50, it was confirmed that gallic acid reacts with Fe(II) ions at a 1:1 ratio, and that the amount of coordination compounds formed increases with the increasing pH. Based on the obtained data, two models were set up in order to obtain additional information about the binding process of gallic acid to Fe(II) ions. Model A revealed an exponential dependence of the apparent formation constant $K_{\mathrm{eq},A}$ on the pH. The more advanced Model B provided an explanation of the observed phenomena, by introducing a twice-deprotonated gallic acid as the only reactive species. Moreover, the formation constant determined using this model ($K_{\mathrm{eq},B}$) is in good agreement with the titrimetrically determined formation constant of Powell and Taylor [37]. An additional confirmation of the validity of the model came from the comparison of the molar fractions of the free and bound gallic acid species preformed by a linear combination of the UV/Vis spectra of the coordination compound and the free gallic acid solution. The basic model assumptions were also confirmed using DFT calculations. Furthermore, DFT calculations facilitated the determination of the most likely iron ion binding position and of the resulting coordination compound structure.

## Figures and Tables

**Table 1:** The results of extrapolations of the Job plot curves for sample sets where $c=\;5\cdot 10^{-4}\;\mathrm{mol}\;\cdot L^{-1}$.

pH	$\epsilon_{\mathrm{extrapolated}}\;(L\cdot {\mathrm{mol}}^{-1}\cdot {\mathrm{cm}}^{-1})$	$x_{\mathrm{GA}}$
4.02	1391.0	0.42
4.51	3783.9	0.48
5.00	3956.0	0.52
5.50	4346.2	0.53
	${\bar{\epsilon }}_{\mathrm{extrapolated}}\;(L\cdot {\mathrm{mol}}^{-1}\cdot {\mathrm{cm}}^{-1})$	
	3369.3	

**Table 2:** Parameters obtained by fitting Model A to the experimental points of the Job plot.

$c\;(\mathrm{mol}\cdot L^{-1})$	pH	$\epsilon_{\mathrm{model}\;A}\;(L\cdot {\mathrm{mol}}^{-1}\cdot {\mathrm{cm}}^{-1})$	$K_{\mathrm{eq},A}$	$\Delta G_{r}^{⦵}(\mathrm{kJ}\cdot {\mathrm{mol}}^{-1})$
	3.52		$1.56\cdot 10^{2}$	−12.5
	4.02		$7.95\cdot 10^{2}$	−16.6
$5\cdot 10^{-4}$	4.51	3476.0	$5.47\cdot 10^{3}$	−21.3
	5.00		$2.38\cdot 10^{4}$	−25.0
	5.50		$9.58\cdot 10^{4}$	−28.4
$2\cdot 10^{-3}$	3.52	3476.0	$7.16\cdot 10^{1}$	−10.6
$1\cdot 10^{-3}$	4.24	3476.0	$1.49\cdot 10^{3}$	−18.1

**Table 3:** Parameters obtained by fitting of Model B (for the reactive species $H_{2}{\mathrm{GA}}^{2-}$) to the experimental points of the Job plot.

pH	$\epsilon_{\mathrm{model}\;B}\;(L\cdot {\mathrm{mol}}^{-1}\cdot \mathrm{cm}\cdot^{-1})$	$K_{\mathrm{eq},B}$	$\Delta G_{r}^{⦵}(\mathrm{kJ}\cdot {\mathrm{mol}}^{-1})$
3.52–5.50	3553.0	$4.34\cdot 10^{7}$	−43.6

## Supplementary Materials

The following are available online at https://www.mdpi.com/article/10.3390/foods10112689/s1, Figure S1: Model B reaction scheme, Figure S2: UV/Vis spectra of gallic acid and of coordination compounds of gallic acid and Fe(II) ions, Figures S3 and S4: Extrapolations of individual curves of the Job plot, Figures S5 and S6: The change in the pH of solutions depending on the molar fraction of gallic acid at different pH values of the acetate buffer, Figure S7: Model function A fitting to the experimental results for additionally measured sets at higher concentrations, Figure S8: Dependence of the formation constant of the coordination compound ${\left[\mathrm{GAFe}(\mathrm{II})\right]}^{2+}$ on the pH of the acetate buffer using a logarithmic scale, Figure S9: Model function B fitting to the experimental results of additionally measured sets at higher concentrations, Figure S10: Dependence of the molar fraction (*x*) of gallic acid present in the free form and gallic acid bound in the coordination compound on $x_{\mathrm{GA}}$ for sample sets with the pH range of 3.52 to 5.00, Figure S11: Dependence of the molar fraction (*x*) of Fe(II) present in the free form and Fe(II) bound in the coordination compound on $x_{\mathrm{GA}}$ for sample sets with the pH range of 3.52 to 5.00, Figure S12: Molar fractions of the individual gallic acid species in the bound and free form at certain solution compositions for the pH range of 3.52 to 5.50, Figure S13: The dependence of the formation constant $K_{\mathrm{eq},A}$ on the pH for Model A and of the apparent formation constant *U* for Model B. The apparent formation constant (U) is for the coordination compound [H${}_{2}$GAFe]. Error bars denote fitting error estimates using bootstrapping method, Figure S14: Comparison of gallic acid spectrum (green dashed line), [H${}_{2}$GAFe] spectrum (blue dashed line) and the spectrum obtained as a linear combination of both spectra (red dashed line) to the experimental spectrum (black line) at different solution compositions at the pH of 5.50, Figure S15: Selected frontier molecular orbitals of the [H${}_{2}$GAFe]${}^{+}$ complex calculated using B3LYP/LANL2DZ,6-311+G(d,p)/CPCM level of theory, Table S1: Standard Gibbs reaction free energies calculated from the formation constants of an individual model, Table S2: The Gibbs free energy difference between high- and low- spin states ($\Delta G^{HS-LS}$) as well as between all investigated complexes and the most stable complex structure 3 (Δ(G−G(3))). The calculations were performed using DFT/LANL2DZ, 6-311+(d,p)/CPCM theoretical models, Table S3: The results of the TDDFT calculations obtained using B3LYP/LANL2DZ, 6-311+G(d,p)/CPCM theoretical model, Table S4: Standard Gibbs formation free energy ($\Delta G_{f}^{⦵}$) in kcal mol${}^{-1}$. Calculations were performed using DFT/LANL2DZ, 6-311+G(d,p)/CPCM theoretical models. Finally, Cartesian coordinates of all structures obtained using DFT calculations are provided.

## Abbreviations

The following abbreviations are used in this manuscript:

CPCM	conductor-like polarizable continuum model
DFT	density functional theory
GA	3,4,5-trihydroxybenzoic acid
HOMO	highest occupied molecular orbital
ILCT	intraligand charge transfer
LMCT	ligand-to-metal charge transition
LUMO	lowest unoccupied molecular orbital
MLCT	metal-to-ligand charge transfer
MOs	molecular orbitals
SOMO	a singly occupied molecular orbital
TDDFT	time-dependent density functional theory
