# Supplementary material for: Dependence of the Fe(II)-Gallic Acid Coordination Compound Formation Constant on the pH
Source: Foods. 2021 Nov 3;10(11):2689. doi: 10.3390/foods10112689 (PMC8619179; doi:10.3390/foods10112689)
Supplement: Supplementary file 1 [file foods-10-02689-s001.zip › foods-1440367-Supplementary material.pdf]

Supplementary Materials: Dependence of the Fe(II)-Gallic Acid Coordination Compound Formation Constant on the pH

Franjo Frešer [1], Gregor Hostnik [1], Jelena Tošović [1] and Urban Bren [1,2,*]

1. Model A - bimolecular reaction

Initially, a simple bimolecular reaction between gallic acid (GA) and Fe(II) in a 1:1 ratio was assumed, as presented in Equation 1. The last step in the equation represents the oxidation of Fe(II) into Fe(III), which takes place after binding of Fe(II) onto gallic acid. Blue coloured solution arises from the gallic acid coordination compound with Fe(III) [1,2]. The formation of gallic acid coordination compound with Fe(III) ion is assumed to be irreversible: the Fe(III) ion bound to gallic acid cannot be exchanged with the Fe(II) ion anymore due to the much larger formation constant with the Fe(III) compared to Fe(II) [3,4].

$$\mathrm{GA} + \mathrm{Fe(II)} \overset{K_{\mathrm{eq,A}}}{\rightleftharpoons} [\mathrm{GAFe(II)}]^{2+} \xrightarrow{\text{oxidation}} [\mathrm{GAFe(III)}]^{3+} \tag{1}$$

($K_{\mathrm{eq,A}}$) denotes the formation constant of the coordination compound $[\mathrm{GAFe(II)}]^{2+}$, which is defined as:

$$K_{\mathrm{eq,A}} = \frac{[[\mathrm{GAFe(II)}]^{2+}]}{[\mathrm{GA}] \cdot [\mathrm{Fe(II)}]}. \tag{2}$$

In the Equation 2 the square brackets denote equilibrium concentrations of a given species. The equilibrium concentration of gallic acid ([GA]) was expressed from the total concentration of gallic acid (c_{GA}) and the equilibrium concentration of the coordination compound formed ($[[\mathrm{GAFe(II)}]^{2+}]$). In an analogous manner, the equilibrium concentration of Fe(II) ions ([Fe(II)]) was expressed from the total concentration of Fe(II) ions ($c_{\mathrm{Fe(II)}}$) and the equilibrium concentration of the coordination compound formed ($[[\mathrm{GAFe(II)}]^{2+}]$).

Because the model was fitted to the Job plot, the molar fraction of gallic acid x_{GA} (Equation 3) was introduced for the calculations and for the construction of the graphs. A new variable c was defined as the sum of total concentrations of gallic acid and Fe(II) ions, and was kept at a constant value of $5 \cdot 10^{-4}$ mol $\cdot$ L^{-1} for all experiments.

$$x_{\mathrm{GA}} = \frac{n_{\mathrm{GA}}}{n_{\mathrm{GA}} + n_{\mathrm{Fe(II)}}} = \frac{c_{\mathrm{GA}}}{c_{\mathrm{GA}} + c_{\mathrm{Fe(II)}}} = \frac{c_{\mathrm{GA}}}{c} \tag{3}$$

Combining the above equations a second-order polynomial was obtained for the equilibrium concentration of the coordination compound formed ($[[\mathrm{GAFe(II)}]^{2+}]$) (the physically meaningful solution of the quadratic equation is achieved by considering the negative sign before the square root). The model value of absorbance (Equation 4) was then expressed by using Beer-Lambert's law:

$$A_{\text{model A}} = [[\mathrm{GAFe(II)}]^{2+}] \cdot \epsilon \cdot b = \frac{1 + K_{\mathrm{eq,A}} \cdot c - \sqrt{1 + 2 \cdot K_{\mathrm{eq,A}} \cdot c + [K_{\mathrm{eq,A}} \cdot c \cdot (1 - 2 \cdot x_{\mathrm{GA}})]^2}}{2 \cdot K_{\mathrm{eq,A}}} \cdot \epsilon \cdot b \tag{4}$$

Variable b stands for the optical path in the absorbance measurement ($b = 1$ cm). The model values of absorbance were then fitted to the experimental values, using the method of least squares, where values of the formation constant ($K_{\mathrm{eq,A}}$) and the absorption coefficient (ϵ) were used as fitting parameters. The latter was kept constant for all pH values, while $K_{\mathrm{eq,A}}$ was determined for each pH value separately.

2. Model B - effect of the protolytic equilibrium

Model B was developed in order to explain the pH dependence of the formation constant, $K_{\mathrm{eq,A}}$, determined by Model A. In Model B it was assumed that different species of gallic acid are in a protolytic equilibrium, and that only one of these species (H_2GA^{2-})

reacts with Fe(II) ions, as depicted in the reaction scheme shown in Figure S1. The final, irreversible oxidation of Fe(II) to Fe(III) in the coordination compound was treated in an analogous way as in the case of Model A. Model functions for reactions where alternative protonation species of gallic acid react individually with Fe(II) ions were also derived and fitted to the experimental data.

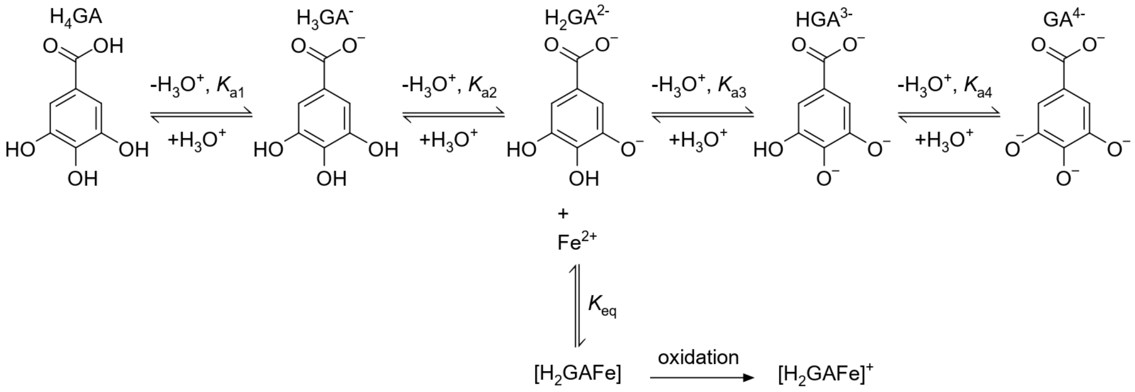

Figure S1. Model B reaction scheme in which H_2GA^{2-} reacts with iron(II) ions. This forms a coordination compound $[H_2GAFe]$, which later irreversibly oxidizes into $[H_2GAFe]^+$.

For the reaction presented in Figure S1 the formation constant is written as:

$$K_{eq,B} = \frac{[[H_2GAFe]]}{[H_2GA^{2-}] \cdot [Fe(II)]}. \quad (5)$$

In Equation 5 variables $[[H_2GAFe]]$, $[H_2GA^{2-}]$, and [Fe(II)] represent equilibrium concentrations of the coordination compound formed, twice deprotonated gallic acid species, and Fe(II) ions, respectively.

The total gallic acid concentration can be written as a sum of the concentrations of individual species (Equation 6). Equation 7 was then obtained by inserting the concentrations of individual species, expressed from the protolytic equilibrium of gallic acid, into Equation 6. The pK_a values of gallic acid determined by Marino *et al.* were used (pK_{a1} = 4.32, pK_{a2} = 8.24, pK_{a3} = 9.97, and pK_{a4} = 13.1) [5].

$$c_{GA} = [H_4GA] + [H_3GA^-] + [H_2GA^{2-}] + [HGA^{3-}] + [GA^{4-}] + [[H_2GAFe]] \quad (6)$$

$$\begin{aligned} c_{GA} &= [H_4GA] \cdot \left(1 + \frac{K_{a1}}{[H_3O^+]} + \frac{K_{a1} \cdot K_{a2}}{[H_3O^+]^2} + \frac{K_{a1} \cdot K_{a2} \cdot K_{a3}}{[H_3O^+]^3} + \frac{K_{a1} \cdot K_{a2} \cdot K_{a3} \cdot K_{a4}}{[H_3O^+]^4}\right) + [[H_2GAFe]] \\ &= [H_4GA] \cdot z + [[H_2GAFe]] \end{aligned} \quad (7)$$

The expression in parentheses was for practical reasons replaced by the variable z, which is a function of the concentration of oxonium ions (pH) and the dissociation constants of gallic acid.

The mass balance for Fe(II) ions in Model B can be written analogously as in the case of Model A. For an easier comparison of the results obtained by Models A and B an apparent equilibrium constant U (Equation 8) was introduced.

$$U_{[H_2GAFe]} = \frac{K_{eq,B} \cdot K_{a1} \cdot K_{a2}}{z \cdot [H_3O^+]^2} \quad (8)$$

Finally, the model function B (Equation 9) was obtained by combining the physically meaningful solution of the quadratic equation with the negative sign before the square root and by applying Beer-Lambert's law:

$$A_{\text{model B}} = [[H_2GAFe]] \cdot \epsilon \cdot b = \frac{1 + U \cdot c - \sqrt{1{+}2 \cdot U \cdot c + [U \cdot c \cdot (1{-}2 \cdot x_{GA})]^2}}{2 \cdot U} \cdot \epsilon \cdot b \quad (9)$$

The model values of absorbance given by Equation 9 were fitted to the experimentally measured values using the method of least squares. In contrast to Model A, the fitting of Model B was performed by taking the same values of $K_{eq,B}$ and ϵ for all pH values. If the Equations 4 and 9 are compared, it can be seen that the variable U in Model B plays an equivalent role as the formation constant $K_{eq,A}$ in Model A.

The model function B, where it is assumed that other protonation species of gallic acid react with Fe(II) ions, is shown by Equations 10 to 13. Their derivation is analogous to the derivation for H_2GA^{2-}.

$$U_{[H_4GAFe]^{2+}} = \frac{K_{eq,B}}{z} \quad (10)$$

$$U_{[H_3GAFe]^{+}} = \frac{K_{eq,B} \cdot K_{a1}}{z \cdot [H_3O^+]} \quad (11)$$

$$U_{[HGAFe]^{-}} = \frac{K_{eq,B} \cdot K_{a1} \cdot K_{a2} \cdot K_{a3}}{z \cdot [H_3O^+]^3} \quad (12)$$

$$U_{[GAFe]^{2-}} = \frac{K_{eq,B} \cdot K_{a1} \cdot K_{a2} \cdot K_{a3} \cdot K_{a4}}{z \cdot [H_3O^+]^4} \quad (13)$$

Figure S2 shows the UV/Vis spectra of gallic acid and gallic acid bound to a coordination compound with Fe(II) ion. It depicts the dependence of the molar absorption coefficient of gallic acid on the wavelength. The black curve, which represents the UV/Vis spectrum of gallic acid (GA), shows the characteristic absorption peaks of gallic acid at wavelengths of 214 and 259 nm. The red curve represents the UV/Vis spectrum of the coordination compound of gallic acid and Fe(II) ion. A wide absorption peak (with maximum value at 568 nm) can be observed in the spectrum due to the coordination compound formation. The intensity of the gallic acid peaks (an equal concentration of gallic acid was used in both solutions) did not change during the coordination compound formation. Both peaks, however, redshifted towards longer wavelengths: the first by 6 and the second by 26 nm.

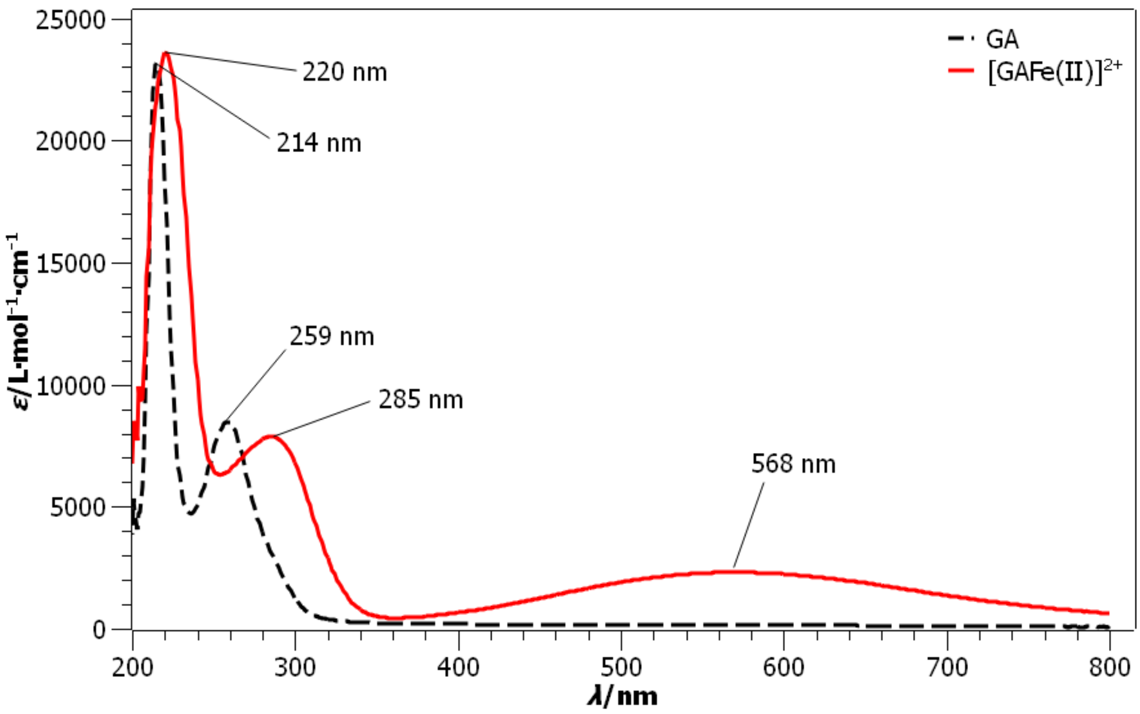

Figure S2. UV/Vis spectra of gallic acid and of coordination compounds of gallic acid and Fe(II) ions. In both $c_{GA} = 5 \cdot 10^{-5}$ mol · L^{-1}, in the coordination compound $c_{Fe(II)} = 5 \cdot 10^{-5}$ mol · L^{-1}; for both solutions pH = 5.50, T = 298.15 K. The noise at wavelengths below 205 nm is due to the buffer absorption. Therefore, the molar absorption coefficients below λ = 230 nm are also burdened with a larger error.

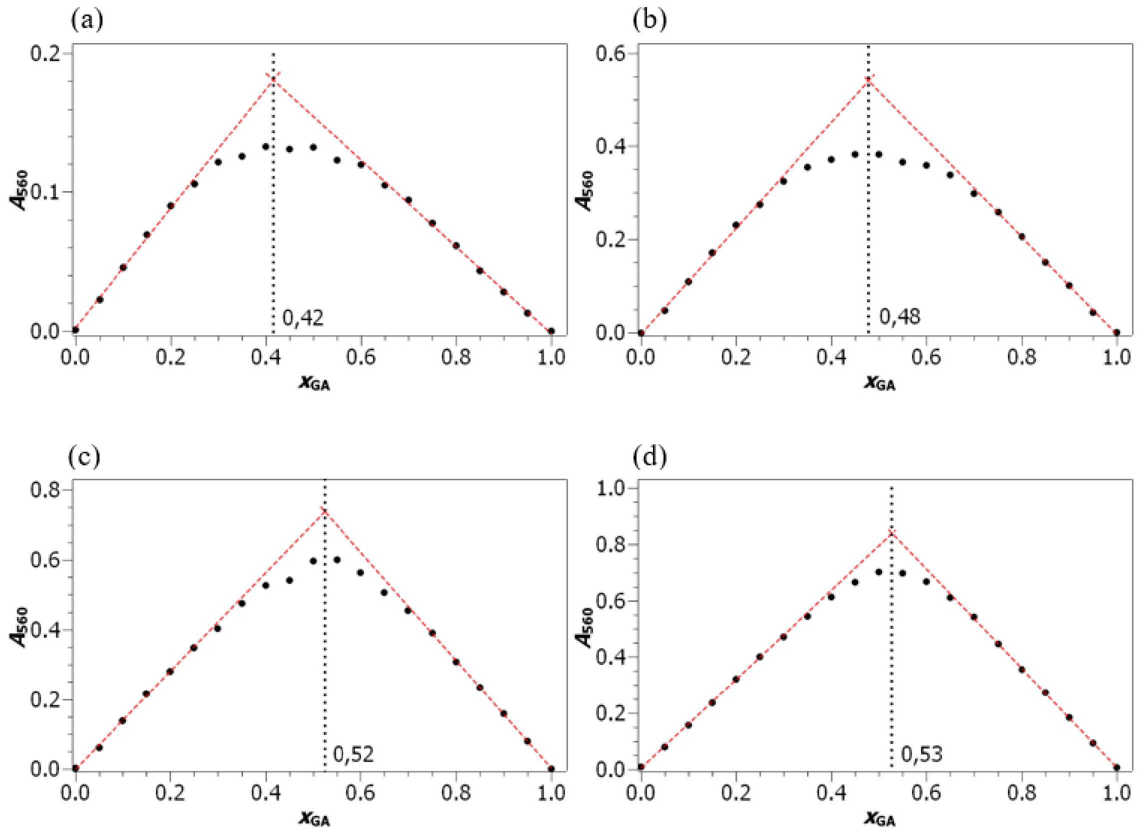

Figure S3. Extrapolations of individual curves of the Job plot, where $c = 5 \cdot 10^{-4}$ mol · L^{-1}: (**a**) pH = 4.02, (**b**) pH = 4.51, (**c**) pH = 5.00, (**d**) pH = 5.50.

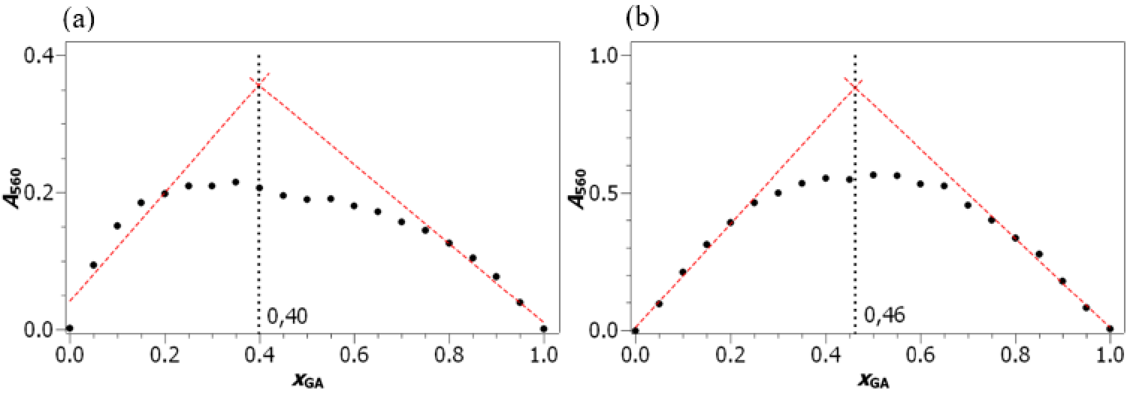

Figure S4. Curves of the Job diagram with extrapolation for additionally measured arrays at higher concentrations: (**a**) pH = 3.52 and $c = 2 \cdot 10^{-3}$ mol · L^{-1}, (**b**) pH = 4.24 and $c = 1 \cdot 10^{-3}$ mol · L^{-1}.

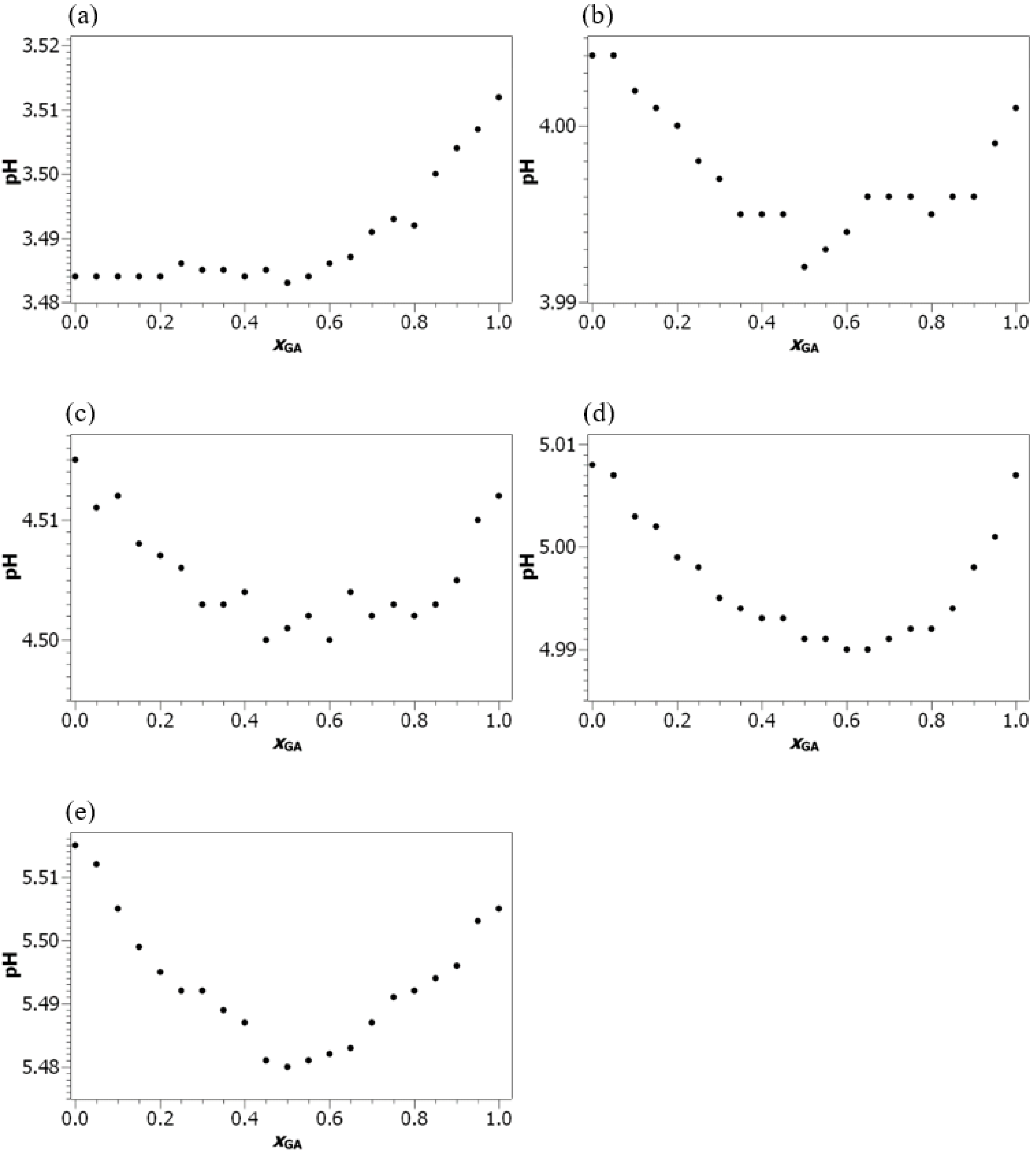

Figure S5. The change in the pH of solutions depending on the molar fraction of gallic acid at different pH values of the acetate buffer ($c = 5 \cdot 10^{-4}$ mol · L^{-1}): (**a**) pH = 3.52, (**b**) pH = 4.02, (**c**) pH = 4.51, (**d**) pH = 5.00, (**e**) pH = 5.50.

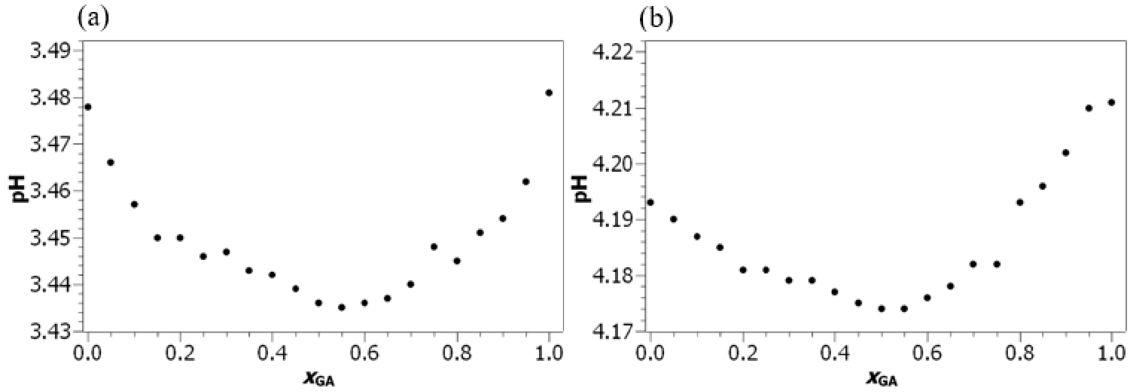

Figure S6. The change in the pH of solutions depending on the molar fraction of gallic acid at different pH values of the acetate buffer for additionally measured arrays at higher concentrations: (**a**) pH = 3.52 and $c = 2 \cdot 10^{-3}$ mol · L^{-1}, (**b**) pH = 4.24 and $c = 1 \cdot 10^{-3}$ mol · L^{-1}.

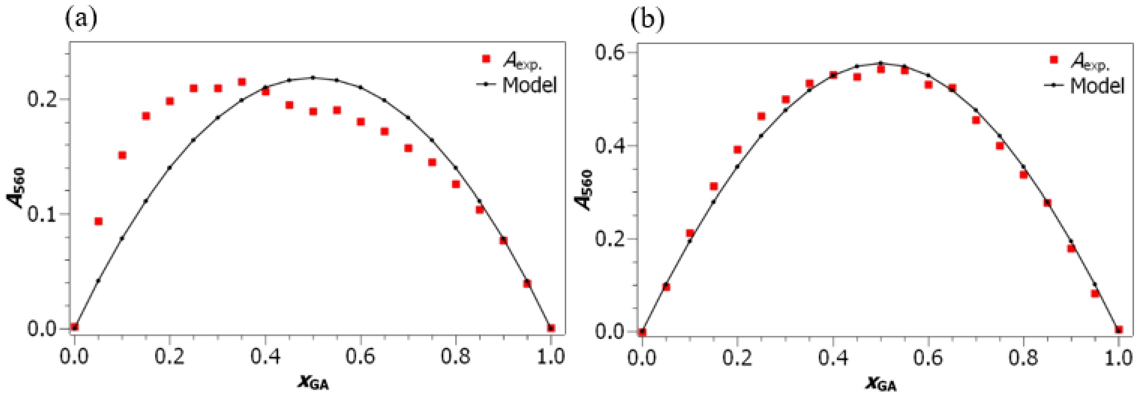

Figure S7. Model function A fitting to the experimental results for additionally measured sets at higher concentrations (using the same ϵ as was determined by the matching for sets with $c = 5 \cdot 10^{-4}$ mol · L^{-1}): (**a**) pH = 3.52 and $c = 2 \cdot 10^{-3}$ mol · L^{-1}, (**b**) pH = 4.24 and $c = 1 \cdot 10^{-3}$ mol · L^{-1}.

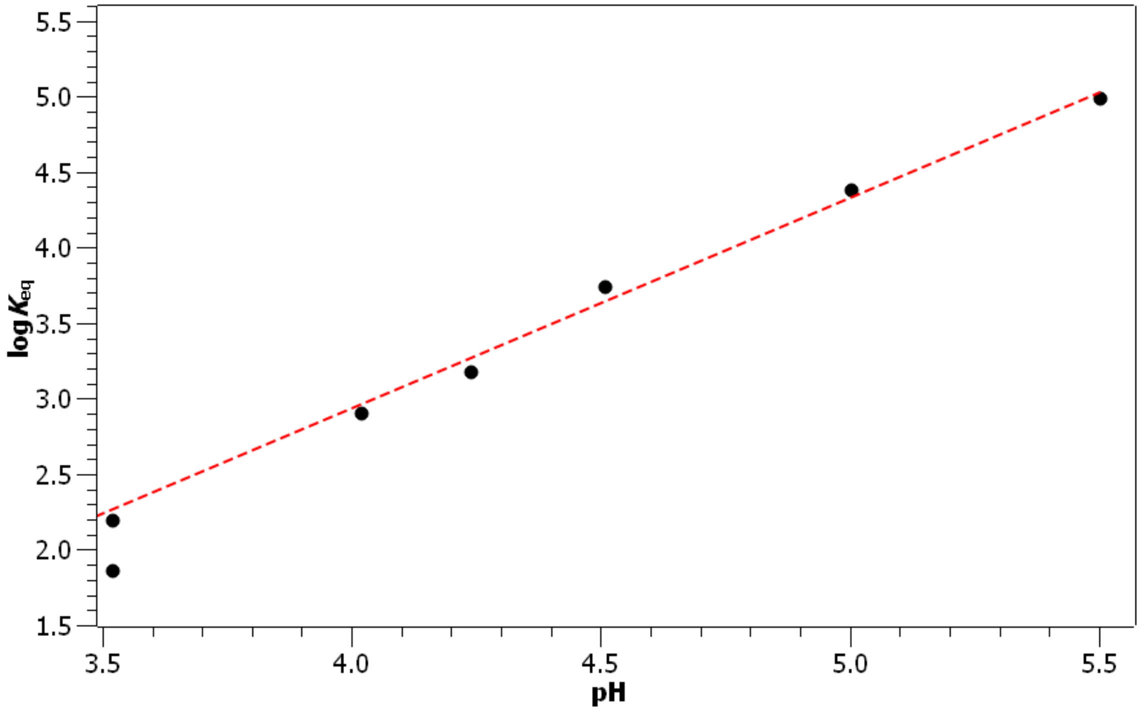

Figure S8. Dependence of the formation constant of the coordination compound $[GAFe(II)]^{2+}$ on the pH of the acetate buffer using a logarithmic scale.

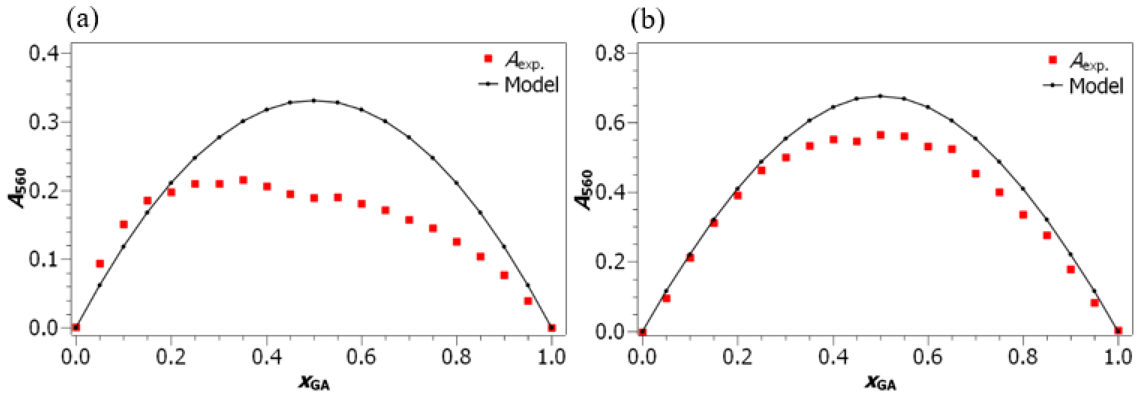

Figure S9. Model function B fitting to the experimental results of additionally measured sets at higher concentrations (using the same ϵ and $K_{eq,B}$ as were determined by the matching for sets with $c = 5 \cdot 10^{-4}$ mol · L^{-1}): (**a**) pH = 3.52 and $c = 2 \cdot 10^{-3}$ mol · L^{-1}, (**b**) pH = 4.24 and $c = 1 \cdot 10^{-3}$ mol · L^{-1}.

Figures S10 and S11 depict the molar fractions calculated using Model B. For comparison, an independent experimental method of fitting the spectra was applied. Thus, UV/Vis spectra from 800 to 200 nm were measured at individual pH values for solutions with different compositions of gallic acid and iron(II) ions. A spectrum of the solution containing an excess of Fe(II) ions and gallic acid was then recorded (thus ensuring that all gallic acid was bound into the coordination compound). This spectrum was then together with the spectrum of gallic acid fitted (linear combination of spectra) to the spectra recorded for solutions with different compositions. It can be observed that the model calculated molar fractions correspond very well to the experimental values (see Figure 8 of the main text).

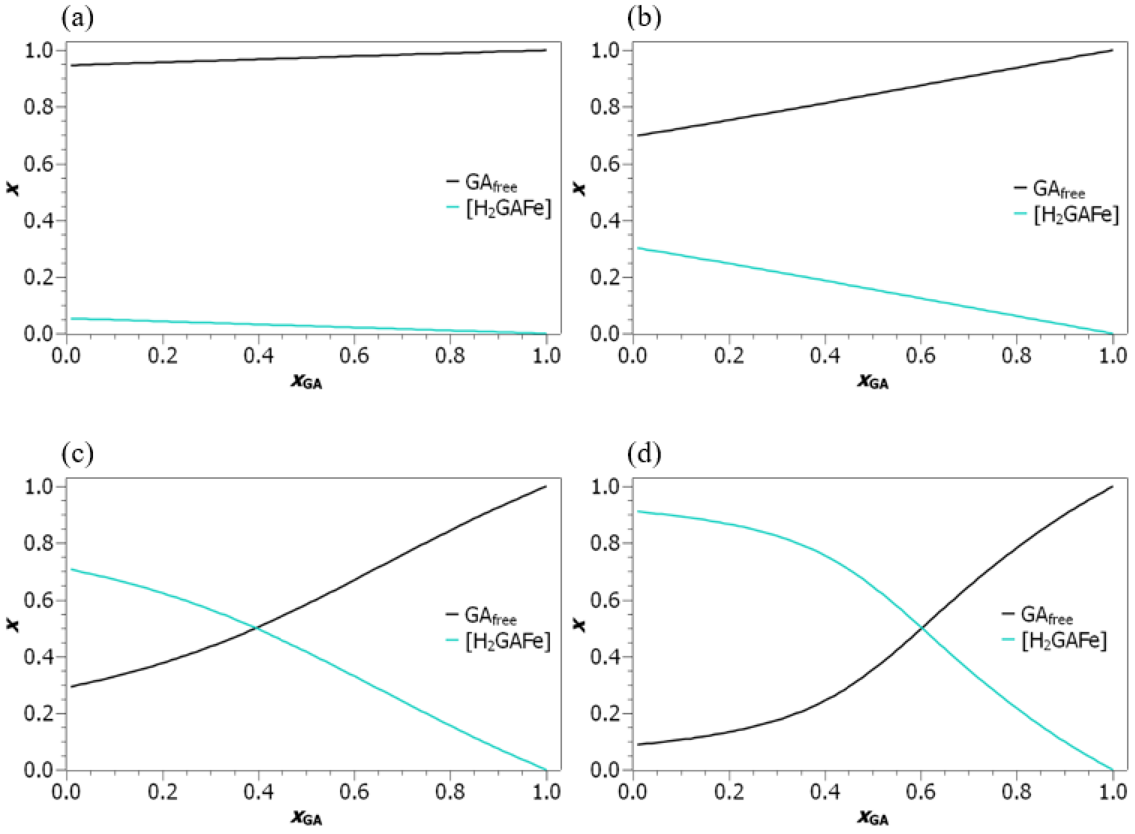

Figure S10. Dependence of the molar fraction (x) of gallic acid present in the free form and gallic acid bound in the coordination compound on x_{GA} for sample sets with: (**a**) pH = 3.52, (**b**) pH = 4.02, (**c**) pH = 4.51, (**d**) pH = 5.00.

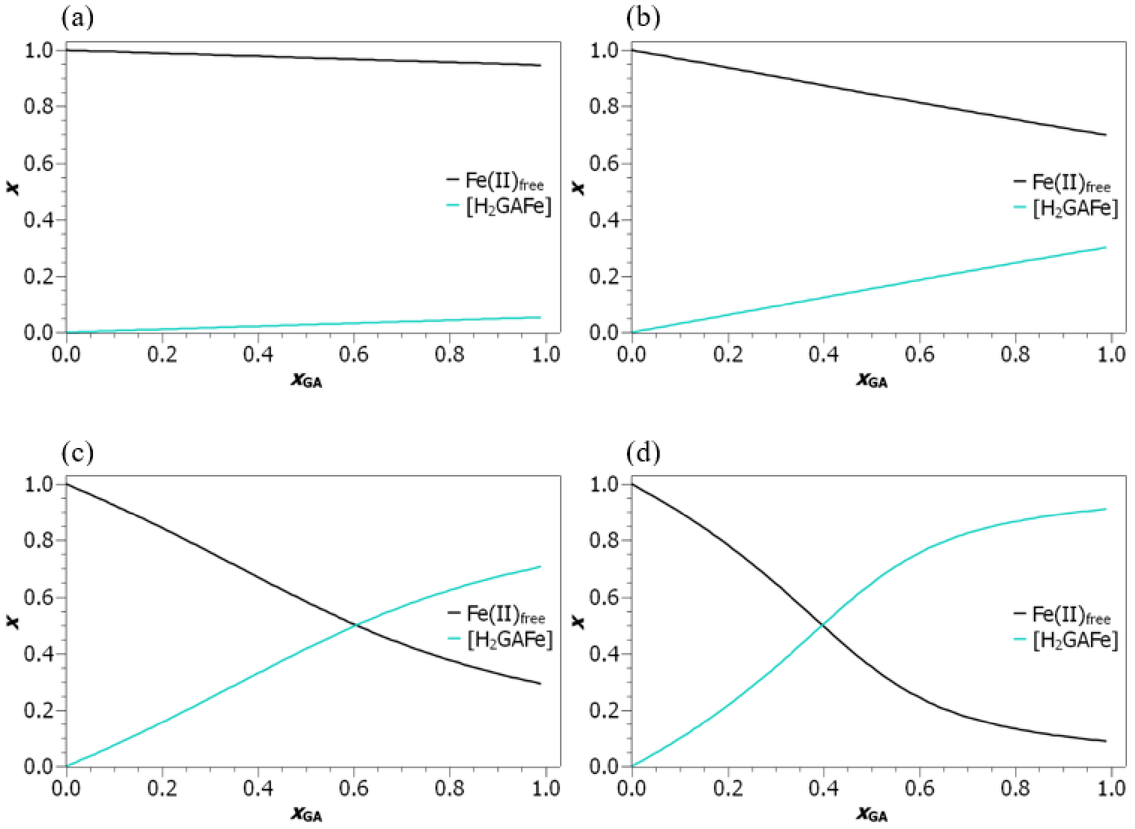

Figure S11. Dependence of the molar fraction (x) of Fe(II) present in the free form and Fe(II) bound in the coordination compound on x_{GA} for sample sets with: (**a**) pH = 3.52, (**b**) pH = 4.02, (**c**) pH = 4.51, (**d**) pH = 5.00.

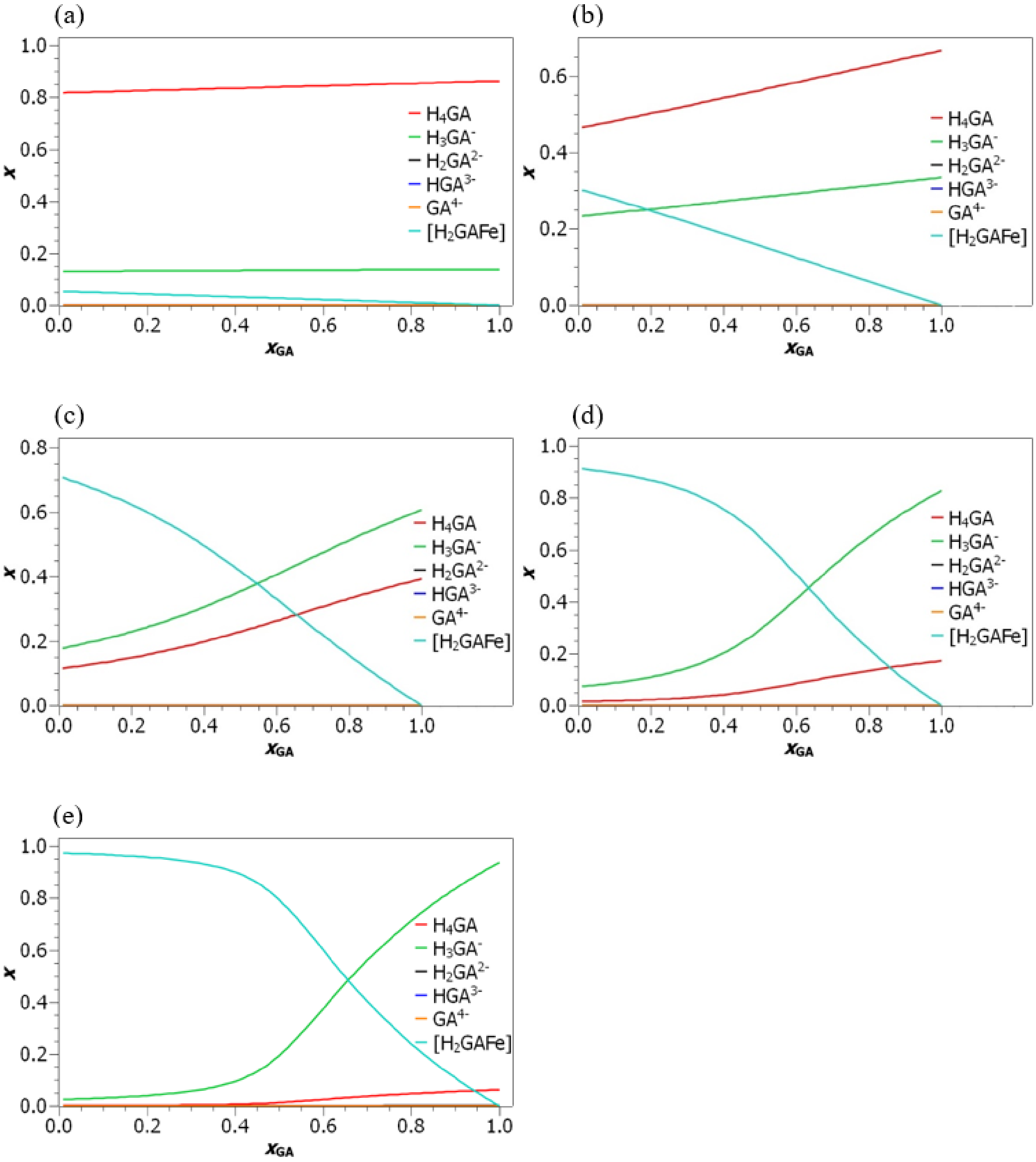

Figure S12. Molar fractions of the individual gallic acid species in the bound and free form at certain solution compositions for the pH range of 3.52 to 5.50: (**a**) pH = 3.52, (**b**) pH = 4.02, (**c**) pH = 4.51, (**d**) pH = 5.00, (**e**) pH = 5.50.

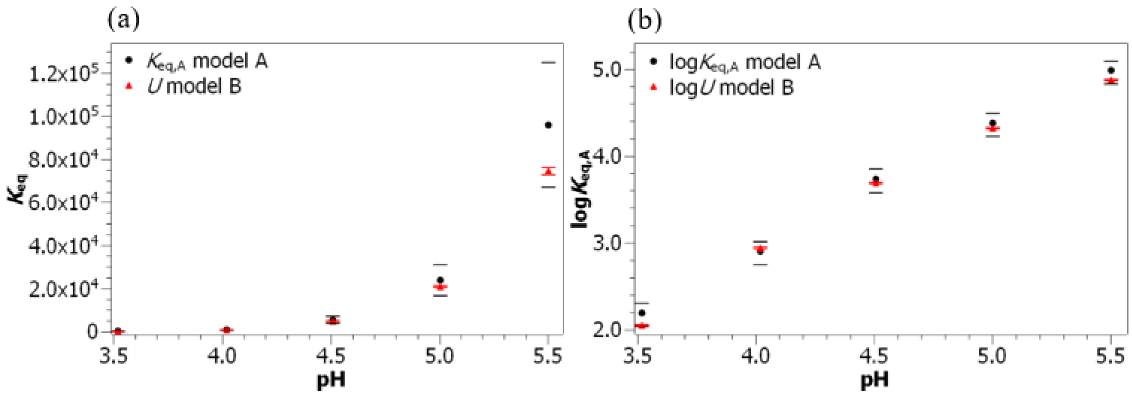

Figure S13. The dependence of the formation constant $K_{eq,A}$ on the pH for Model A and of the apparent formation constant U for Model B which were determined for sets with $c = 5 \cdot 10^{-4}$ mol · L^{-1}. The apparent formation constant (U) is for the coordination compound [H_2GAFe]. Using: (**a**) ordinary scale, (**b**) logarithmic scale. Error bars denote fitting error estimates using a bootstrapping method.

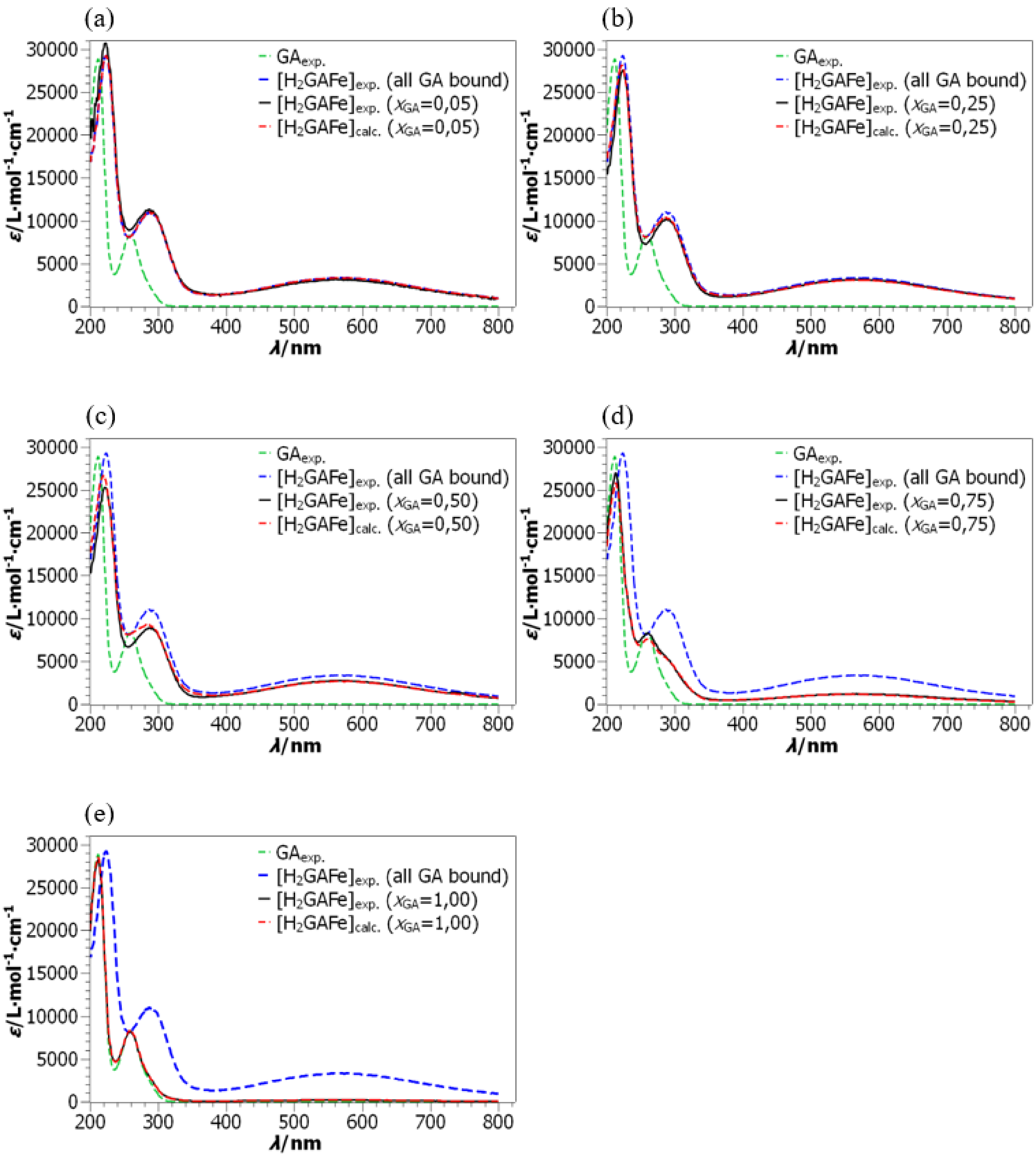

Figure S14. Comparison of gallic acid spectrum (green dashed line), [H_2GAFe] spectrum (blue dashed line) and the spectrum obtained as a linear combination of both spectra (red dashed line) to the experimental spectrum (black line) at (**a**) $x_{GA} = 0.05$, (**b**) $x_{GA} = 0.25$, (**c**) $x_{GA} = 0.50$, (**d**) $x_{GA} = 0.75$ and (**e**) $x_{GA} = 1.00$ at the pH of 5.50.

Table S1: Standard Gibbs reaction free energies calculated from the formation constants of an individual model.

pH	$\Delta G^{\ominus}_{\text{r, model A}}(\text{kJ} \cdot \text{mol}^{-1})$	$\Delta G^{\ominus}_{\text{r, model B}}(\text{kJ} \cdot \text{mol}^{-1})$
3.52	-12.5	-11.7
4.02	-16.6	-16.8
4.51	-21.3	-21.1
5.00	-25.0	-24.7
5.50	-28.4	-27.8

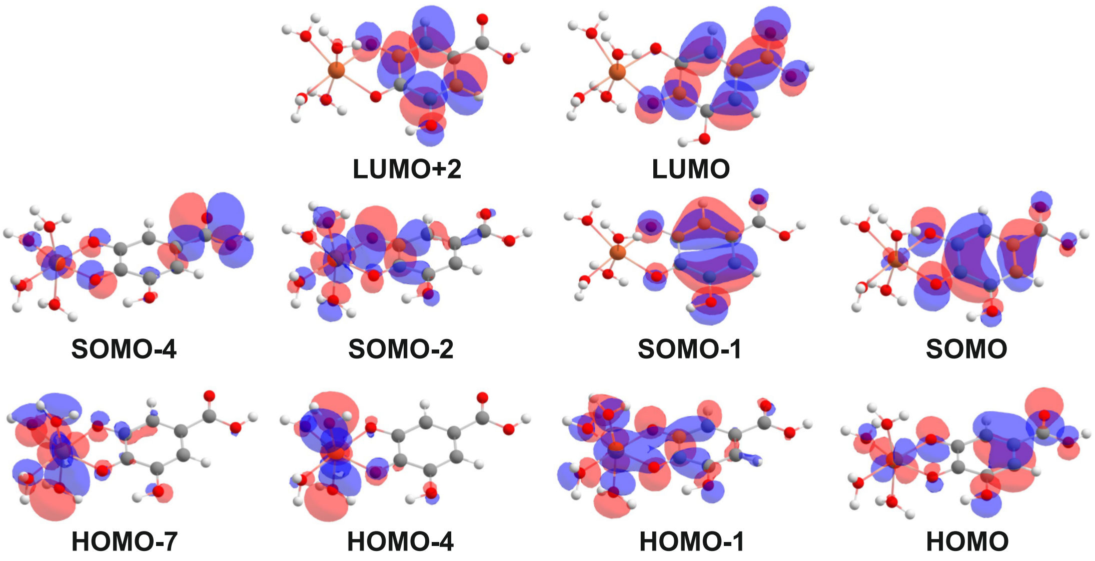

Figure S15. Selected frontier molecular orbitals of the $[H_2GAFe]^+$ complex calculated using B3LYP/LANL2DZ,6-311+G(d,p)/CPCM level of theory. (Isosurface value = 0.03 e/au^3)

Table S2: The Gibbs free energy difference between high- and low- spin states (ΔG^{HS-LS}) as well as between all investigated complexes and the most stable complex structure 3 ($\Delta(G - G(3))$) in kcal·mol^{-1}. The calculations were performed using the DFT/LANL2DZ, 6-311+(d,p)/CPCM theoretical models.

Method	B3LYP		M06	ωB97XD
Structure	ΔG^{HS-LS}	$\Delta(G - G(3))$		
	$[H_2GAFe]$			
1	-32.0	11.7	10.5	10.3
2	-32.0	6.0	6.4	5.3
3	-32.2	0.0	0.0	0.0
4	-34.2	10.2	9.3	8.9
5	-35.7	11.5	10.9	10.3
6	-35.0	30.3	28.4	25.3
	$[H_2GAFe]^+$			
1	-20.9	11.4	12.4	9.4
2	-32.8	6.4	5.8	5.0
3	-27.9	0.0	0.0	0.0
4	-29.8	20.9	20.4	20.4
5	-33.8	26.4	24.9	24.7
6	-35.6	32.2	32.9	30.8
	$[H_3GAFe]^+$			
7	-32.9	0.0	0.0	0.0
8	-37.0	20.2	20.9	18.9

Table S3: The results of the TDDFT calculations obtained using the B3LYP/LANL2DZ, 6-311+G(d,p)/CPCM theoretical model.

λ^{exp}_{max}	λ^{calc}_{max}	f	ΔE	MO description	Type
568	539.8	0.0632	1.8831	(67 69) HOMO-1→SOMO-4 (97.3 %)	MLCT/ILCT
	300.8	0.0572	3.3797	(68 71) HOMO → SOMO-2 (78.6 %)	LMCT/ILCT
285	292.3	0.0470	3.4776	(73 74) SOMO → LUMO (58.7 %)	ILCT
	268.3	0.0353	3.7895	(64 69) HOMO-4→SOMO-4 (77.6 %)	MLCT
	228.1	0.0399	4.4571	(72 76) SOMO-1 →LUMO+2 (51.8 %)	ILCT
220	220.4	0.0411	4.6120	(61 69) HOMO-7→SOMO-4 (62.4%)	MLCT
	214.8	0.0396	4.7333	(67 73) HOMO-1→SOMO (83.0%)	MLCT/ILCT

* Note: λ_{max} absorption wavelength in nm. ΔE excitation energies in eV. f, oscillator strengths. MO description, molecular orbital approximation of each electron excitation. Experimental λ_{max} values in nm are given for comparison. HOMO, SOMO, and LUMO denote the highest-, singly- and lowest-(un)occupied molecular orbitals, respectively. MLCT, ILCT and LMCT denote metal-to-ligand, intra ligand, and ligand-to-metal charge transfer, respectively.

Table S4: Standard Gibbs formation free energy ($\Delta G_f^{\ominus}$) in kcal mol^{-1}. Calculations were performed using the DFT/LANL2DZ, 6-311+G(d,p)/CPCM theoretical models.

	B3LYP	M06	ωB97XD
$[H_3GAFe]^+$	-20.2	-19.4	-18.3
$[H_2GAFe]$	-47.6	-46.2	-46.7
$[H_2GAFe]^+$	-119.4	-110.7	-120.8

References

1. Jancovicova, V.; Ceppan, M.; Havlinova, B.; Rehakova, M.; Jakubikova, Z. Interactions in iron gall inks. *Chem. Zvesti* **2007**, *61*, 391–397. doi:{10.2478/s11696-007-0053-0}.
2. Lutui, M.A.; Gilard, F.; Sablier, M. Can electrospray ionization help the characterization of iron gall inks? Investigation on the interactions of gallic acid with iron ions in aqueous solutions. *J. Mass Spectrom.* **2008**, *43*, 1123–1131. doi:{10.1002/jms.1396}.
3. Powell, H.; Taylor, M. Interactions of iron(II) and iron(III) with gallic acid and its homologues - a potentiometric and spectrophotometric study. *Aust. J. Chem.* **1982**, *35*, 739–756. doi:{10.1071/CH9820739}.
4. Perron, N.R.; Brumaghim, J.L. A Review of the Antioxidant Mechanisms of Polyphenol Compounds Related to Iron Binding. *Cell Biochem. Biophys.* **2009**, *53*, 75–100. doi:{10.1007/s12013-009-9043-x}.
5. Marino, T.; Galano, A.; Russo, N. Radical Scavenging Ability of Gallic Acid toward OH and OOH Radicals. Reaction Mechanism and Rate Constants from the Density Functional Theory. *J. Phys. Chem. B.* **2014**, *118*, 10380–10389. doi:{10.1021/jp505589b}.

Cartesian coordinates in Å for all investigated complexes of iron and gallic acid calculated using the B3LYP/LANL2DZ,6-311+G(d,p) theoretical model.

Complex 1

0 1	x	y	z
Fe	-2.573697	0.004545	0.023305
O	-2.652331	-0.230808	-2.000976
H	-2.743788	0.572047	-2.530659
O	-4.065293	1.411497	0.045594
H	-4.937174	1.082851	-0.210913
O	-4.095617	-1.367354	0.126644
H	-4.129395	-1.862454	0.955666
O	-2.666976	0.067263	2.054292
H	-1.842309	-0.098466	2.529467
H	-3.018921	0.902014	2.391732
H	-4.125203	-2.015249	-0.589234
H	-1.88497	-0.701604	-2.353892
H	-3.936069	2.254715	-0.40634
C	1.274374	0.05369	-0.008888
C	1.955068	1.282161	-0.090306
C	3.366446	1.329093	-0.102521
C	4.031862	0.064734	-0.028115
C	3.360916	-1.145281	0.052196
C	1.966076	-1.164883	0.062673
H	1.392532	2.206305	-0.144408
H	1.434571	-2.104656	0.125262
C	-0.195665	0.037777	0.001653
O	-0.868274	-1.057029	0.072013
O	-0.894904	1.110344	-0.05798
O	4.056333	-2.334566	0.122018
H	5.001657	-2.135839	0.103366
O	5.39963	0.102348	-0.039749
H	5.578238	1.067762	-0.101618
O	4.115844	2.38517	-0.17347

Complex 1

0 5	x	y	z
Fe	-2.648526	0.073655	-0.030537
O	-2.872225	-0.416051	-2.172266
H	-3.356836	0.106672	-2.822902
O	-4.012835	1.752641	-0.158354
H	-4.292521	2.278108	0.601022
O	-4.145613	-1.466504	0.082069
H	-4.22824	-1.97466	0.898885
O	-2.808052	-0.139274	2.165356
H	-2.028031	-0.509433	2.597483
H	-3.17882	0.509539	2.77596

H	-4.231514	-2.07846	-0.659772
H	-2.10034	-0.775467	-2.627222
H	-4.042026	2.339423	-0.923901
C	1.348827	0.03826	-0.00875
C	2.002025	1.280788	-0.100014
C	3.412078	1.364141	-0.094027
C	4.108615	0.119953	0.009219
C	3.466791	-1.104781	0.098879
C	2.072518	-1.159476	0.091071
H	1.417699	2.189506	-0.175352
H	1.56359	-2.111179	0.161188
C	-0.12875	-0.018137	-0.015415
O	-0.759851	-1.118274	0.069926
O	-0.8193	1.067102	-0.107446
O	4.191989	-2.275235	0.196605
H	5.131793	-2.051332	0.187867
O	5.476342	0.193073	0.016036
H	5.629042	1.162097	-0.057567
O	4.135133	2.439432	-0.171231

Complex 2

0 1	x	y	z
Fe	-2.571637	0.001522	0.09537
O	-2.689144	-0.119813	-1.938813
H	-2.780605	0.713729	-2.418762
O	-4.057395	1.413226	0.228041
H	-4.9354	1.102492	-0.029685
O	-4.102495	-1.368408	0.150678
H	-4.120369	-1.911421	0.94958
O	-2.622009	-0.052782	2.129941
H	-1.782553	-0.243833	2.568369
H	-2.958883	0.764529	2.521106
H	-4.147639	-1.97358	-0.600827
H	-1.922211	-0.568376	-2.320719
H	-3.932005	2.278184	-0.181796
C	1.259323	0.034602	-0.011113
C	1.959886	1.260903	-0.03371
C	3.33878	1.258911	-0.069547
C	4.096672	0.052953	-0.085078
C	3.354728	-1.162663	-0.06147
C	1.975983	-1.182466	-0.025606
H	1.415121	2.197045	-0.022737
H	1.443618	-2.12555	-0.00822
C	-0.193019	0.025205	0.026975
O	-0.877835	-1.070686	0.047239
O	-0.895292	1.10466	0.043717
O	4.0986	-2.319305	-0.076628
H	5.022303	-2.008743	-0.101616
O	5.386715	0.061417	-0.118662
O	4.067589	2.424949	-0.09259

H	4.995244	2.126133	-0.115582

Complex 2

0 5	x	y	z
Fe	-2.638774	0.071756	0.049613
O	-2.916569	-0.299795	-2.115214
H	-3.374116	0.285278	-2.731457
O	-4.007334	1.757343	0.044674
H	-4.272295	2.235813	0.83951
O	-4.15895	-1.454282	0.10477
H	-4.242323	-2.008152	0.89099
O	-2.768377	-0.261729	2.237156
H	-1.987264	-0.684562	2.615948
H	-3.077649	0.377969	2.890163
H	-4.276837	-2.016535	-0.671033
H	-2.158009	-0.660975	-2.59077
H	-4.044855	2.391049	-0.682054
C	1.337529	0.016082	-0.010654
C	2.006411	1.259135	-0.045752
C	3.385908	1.295459	-0.063904
C	4.177042	0.112599	-0.0487
C	3.468187	-1.121541	-0.013525
C	2.089479	-1.17895	0.005571
H	1.436296	2.179696	-0.057751
H	1.582469	-2.135719	0.03272
C	-0.122346	-0.03608	0.010288
O	-0.764308	-1.138091	0.048582
O	-0.818527	1.057187	-0.007963
O	4.244609	-2.257628	0.000842
H	5.159143	-1.920251	-0.017842
O	5.46834	0.155933	-0.065698
O	4.081715	2.482443	-0.097524
H	5.017473	2.20938	-0.103131

Complex 3

0 1	x	y	z
Fe	-2.189228	-0.197825	-0.093221
O	-2.260261	-0.229583	1.956551
H	-1.565165	-0.857797	2.203959
O	-3.534431	-1.779366	-0.103104
H	-4.142612	-1.778325	-0.853083
O	-3.737293	1.183581	-0.228182
H	-3.366206	2.072296	-0.144906
O	-2.144341	-0.357072	-2.136191
H	-1.921618	0.480893	-2.563871
H	-1.400406	-0.951553	-2.313264
H	-4.451497	1.130087	0.419421
H	-1.988174	0.621076	2.327903
H	-4.079532	-1.872093	0.688378
C	2.906282	-0.278038	0.057712

C	2.727349	1.119155	0.018084
C	1.439977	1.630609	-0.03126
C	0.316477	0.785767	-0.042478
C	0.492364	-0.628622	-0.002131
C	1.789557	-1.140904	0.047498
H	3.574799	1.791317	0.025574
H	1.940898	-2.213293	0.078402
O	-0.617145	-1.374496	-0.015112
O	-0.908815	1.291289	-0.089609
O	1.218063	2.989806	-0.07104
C	4.242751	-0.8718	0.109865
O	4.497107	-2.068568	0.146234
O	5.256682	0.041606	0.116022
H	6.085821	-0.457912	0.151475
H	0.253399	3.095366	-0.101639

Complex 3

0 5	x	y	z
Fe	-2.132016	-0.120636	-0.126353
O	-2.535626	0.255473	2.066063
H	-2.578197	-0.47244	2.698137
O	-3.461276	-1.941133	-0.334147
H	-4.330553	-1.910165	0.084249
O	-4.012331	0.996476	-0.202011
H	-4.225038	1.478603	-1.01035
O	-2.532231	-0.038322	-2.339699
H	-1.849225	0.303797	-2.929087
H	-2.802462	-0.892195	-2.700581
H	-4.24263	1.558789	0.54752
H	-1.817639	0.831395	2.360868
H	-3.054036	-2.766948	-0.044001
C	3.063676	-0.292611	0.085115
C	2.933228	1.109849	0.151915
C	1.66628	1.668621	0.144341
C	0.506726	0.871561	0.071619
C	0.638854	-0.550678	0.003786
C	1.915303	-1.110718	0.010984
H	3.804854	1.748283	0.207891
H	2.027866	-2.187132	-0.040767
O	-0.494607	-1.2527	-0.065144
O	-0.689258	1.423445	0.068073
O	1.488294	3.033393	0.207311
C	4.376297	-0.935397	0.091582
O	4.589529	-2.140088	0.036057
O	5.423088	-0.061886	0.167951
H	6.233054	-0.592971	0.163861
H	0.524875	3.161932	0.182805

Complex 4

0 1	x	y	z

Fe	2.190673	-0.268566	0.015413
O	2.243395	-0.234503	-2.015623
H	2.371771	-1.077894	-2.469255
O	3.515181	-1.841448	-0.070929
H	3.366693	-2.636236	0.45683
O	3.844707	0.912578	-0.042678
H	3.948077	1.517312	0.703897
O	2.261729	-0.127535	2.0453
H	1.620327	0.53995	2.328594
H	2.075288	-0.923897	2.560026
H	3.904601	1.447626	-0.84544
H	1.493232	0.205437	-2.436762
H	4.424677	-1.561537	0.099888
C	-3.003497	-0.189315	-0.014801
C	-2.733172	1.189176	-0.027296
C	-1.427864	1.656983	-0.005994
C	-0.336293	0.765381	0.022661
C	-0.640624	-0.598787	0.042657
C	-1.933506	-1.093008	0.027405
H	-3.557398	1.89092	-0.055919
H	-2.129374	-2.158063	0.047888
O	0.509826	-1.420684	0.11992
O	0.91622	1.214285	0.027825
O	-1.151839	3.001904	-0.024769
C	-4.44143	-0.700982	-0.038221
O	-4.604385	-1.952855	-0.019562
O	-5.359222	0.164465	-0.074588
H	-0.18437	3.079789	-0.012299
H	0.403092	-2.233324	-0.391525

Complex 4

0 5	x	y	z
Fe	2.237097	0.065248	0.038471
O	2.597646	0.02083	-2.181218
H	2.65506	-0.866362	-2.558057
O	3.352598	-1.828558	-0.149696
H	3.060522	-2.644192	0.275834
O	4.277973	0.851129	-0.024941
H	4.671044	1.385429	0.675922
O	2.457135	-0.170774	2.227251
H	2.424642	0.621062	2.778636
H	1.87846	-0.816866	2.651554
H	4.545774	1.240825	-0.866988
H	1.975363	0.511209	-2.73272
H	4.291525	-1.732287	0.057309
C	-3.183464	-0.251719	-0.008673
C	-2.952971	1.129517	-0.074177
C	-1.655796	1.626329	-0.080628
C	-0.547483	0.763915	-0.023166
C	-0.803105	-0.610866	0.043701

C	-2.089365	-1.127506	0.051047
H	-3.794088	1.809596	-0.119837
H	-2.263987	-2.195574	0.103628
O	0.356535	-1.378829	0.107156
O	0.692785	1.253232	-0.035199
O	-1.414186	2.976237	-0.146141
C	-4.608769	-0.801395	0.003968
O	-4.738338	-2.055765	0.064168
O	-5.549416	0.038274	-0.044871
H	-0.448609	3.077154	-0.136361
H	0.161932	-2.322122	0.05667

Complex 5

0 1	x	y	z
Fe	2.210664	-0.182524	-0.033161
O	2.386654	-0.121124	-2.066143
H	2.504387	-0.996481	-2.458962
O	3.313902	-1.884636	-0.09349
H	2.786314	-2.65248	0.165755
O	3.974083	0.888018	-0.021811
H	4.099806	1.614097	0.6019
O	2.169005	-0.274183	2.005556
H	2.051284	0.543528	2.506778
H	1.426144	-0.848444	2.242872
H	4.196323	1.234228	-0.896502
H	1.649754	0.292918	-2.534554
H	4.105218	-1.889168	0.461085
C	-2.940887	-0.239678	0.032268
C	-2.790168	1.154649	0.002003
C	-1.513431	1.707923	-0.055545
C	-0.410845	0.866607	-0.075972
C	-0.517842	-0.533345	-0.033456
C	-1.817472	-1.071058	0.011909
H	-3.666287	1.790974	0.022329
H	-1.946102	-2.145494	0.043366
O	0.593066	-1.263424	-0.027408
O	0.900622	1.363873	-0.166055
O	-1.245378	3.056374	-0.086671
C	-4.350401	-0.845741	0.092431
O	-4.433507	-2.10309	0.113727
O	-5.318026	-0.037383	0.116824
H	-2.063138	3.558137	0.012084
H	0.973886	2.259383	0.192243

Complex 5

0 5	x	y	z
Fe	2.187066	-0.4253	0.032901
O	2.498065	-0.134034	-2.150499
H	2.554827	-0.888668	-2.749761
O	3.508037	-2.139816	0.108267

H	3.960271	-2.26539	0.952145
O	3.872688	0.957988	0.039981
H	3.944062	1.730294	0.614351
O	2.504202	-0.612083	2.263694
H	2.716978	0.150104	2.816781
H	1.742266	-1.034306	2.681701
H	4.137695	1.239082	-0.845555
H	1.900303	0.498494	-2.569377
H	4.103874	-2.448751	-0.584601
C	-3.115965	-0.16961	0.004233
C	-2.954481	1.220576	-0.074306
C	-1.6733	1.765927	-0.103208
C	-0.56696	0.928435	-0.051608
C	-0.694953	-0.467623	0.038186
C	-1.992367	-1.001164	0.061925
H	-3.828235	1.859971	-0.113901
H	-2.124301	-2.07361	0.124141
O	0.412702	-1.207549	0.093778
O	0.732749	1.408498	-0.088416
O	-1.395614	3.11312	-0.183502
C	-4.526451	-0.771032	0.018908
O	-4.61404	-2.02847	0.049852
O	-5.492676	0.039479	-0.004043
H	-2.213632	3.622965	-0.205386
H	0.74482	2.374931	-0.103281

Complex 6

0 1	x	y	z
Fe	2.224986	-0.249365	0.043448
O	2.177178	-0.231123	-1.976689
H	2.403234	-1.07251	-2.39675
O	3.477651	-1.828982	-0.122807
H	3.319989	-2.654858	0.353041
O	3.885107	0.887019	-0.09563
H	4.098752	1.454714	0.657006
O	2.421411	-0.137989	2.058377
H	1.80786	0.486124	2.470583
H	2.362571	-0.95674	2.568992
H	3.958151	1.425985	-0.895
H	1.38697	0.104005	-2.420903
H	4.405011	-1.592273	0.018185
C	-3.002206	-0.175285	-0.053097
C	-2.762484	1.205085	-0.077977
C	-1.450942	1.746236	-0.008445
C	-0.430251	0.770533	0.099641
C	-0.653418	-0.583432	0.111778
C	-1.942634	-1.095246	0.043827
H	-3.606754	1.878836	-0.161522
H	-2.140361	-2.15838	0.061952
O	0.519738	-1.36775	0.216591

O	0.880495	1.237098	0.164608
O	-1.089099	2.988236	-0.048136
C	-4.440268	-0.709158	-0.131908
O	-4.585811	-1.962537	-0.108343
O	-5.368155	0.14049	-0.214446
H	0.821461	2.208115	0.022316
H	0.411357	-2.226012	-0.21481

Complex 6

0 5	x	y	z
Fe	2.297991	-0.221671	-0.000386
O	2.377926	-0.176042	-2.133751
H	2.595462	-0.982266	-2.618964
O	3.581594	-1.937521	-0.308039
H	3.393641	-2.788346	0.107802
O	3.902011	1.182128	0.34925
H	3.847539	2.093866	0.036741
O	2.354096	-0.3816	2.131827
H	2.754846	0.33072	2.646598
H	1.604316	-0.721323	2.636601
H	4.839909	0.958675	0.39623
H	1.743012	0.323278	-2.66277
H	4.539693	-1.887581	-0.415418
C	-3.112885	-0.186355	0.013389
C	-2.880812	1.190796	-0.101543
C	-1.568548	1.728976	-0.142771
C	-0.533206	0.765615	-0.058738
C	-0.750161	-0.586596	0.055644
C	-2.043407	-1.095574	0.095826
H	-3.729902	1.860839	-0.162261
H	-2.235752	-2.156404	0.187734
O	0.418296	-1.359774	0.131449
O	0.764667	1.260795	-0.106289
O	-1.210428	2.97106	-0.249346
C	-4.549701	-0.725199	0.05125
O	-4.689645	-1.975043	0.161324
O	-5.485459	0.116391	-0.031223
H	0.631687	2.236086	-0.188252
H	0.232763	-2.307096	0.141969

Complex 1

1 2	x	y	z
Fe	-2.855056	-0.318491	-0.218148
O	-2.82021	0.41642	-2.141545
H	-3.221366	1.29163	-2.238502
O	-3.449523	1.764065	0.352969
H	-4.223968	1.881299	0.91742
O	-4.87013	-0.695716	-0.371447
H	-5.186477	-1.555064	-0.063383
O	-2.848565	-1.422079	1.465768

H	-1.864019	-1.478313	1.686089
H	-3.331467	-1.103354	2.238158
H	-5.352013	-0.490135	-1.183123
H	-1.907488	0.497252	-2.453256
H	-2.773726	2.364332	0.692474
C	1.401045	-0.256989	0.223929
C	1.724443	0.651184	-0.754083
C	3.10692	0.946535	-1.031611
C	4.114248	0.241195	-0.250085
C	3.760895	-0.679693	0.735367
C	2.415502	-0.919713	0.971606
H	0.961515	1.158989	-1.327729
H	2.12818	-1.628677	1.73681
C	-0.034853	-0.578264	0.534195
O	-0.292328	-1.322659	1.503865
O	-0.910232	-0.047914	-0.241684
O	4.701865	-1.341556	1.46537
H	5.585654	-1.07294	1.179824
O	5.403208	0.493068	-0.494024
H	5.43533	1.157421	-1.209415
O	3.50558	1.764294	-1.898995

Complex 1

1 6	x	y	z
Fe	-2.67252	0.075068	-0.030184
O	-2.874128	-0.400354	-2.161533
H	-3.414274	0.078144	-2.802476
O	-4.012094	1.756358	-0.154834
H	-4.292058	2.284737	0.602596
O	-4.119704	-1.494716	0.085164
H	-4.197234	-2.006259	0.900528
O	-2.792654	-0.140922	2.15232
H	-2.013849	-0.474852	2.614764
H	-3.246161	0.451496	2.764442
H	-4.212678	-2.102124	-0.659696
H	-2.11233	-0.745672	-2.643398
H	-4.068274	2.330242	-0.928763
C	1.348366	0.093456	-0.014821
C	1.980923	1.309072	-0.108858
C	3.419722	1.371197	-0.101161
C	4.144725	0.11179	0.009692
C	3.478742	-1.110177	0.104432
C	2.092045	-1.115821	0.091478
H	1.421231	2.230937	-0.189012
H	1.563121	-2.057053	0.164117
C	-0.143488	0.019222	-0.020268
O	-0.732446	-1.093882	0.061887
O	-0.824776	1.096392	-0.106806
O	4.156749	-2.286959	0.208408
H	5.108639	-2.117559	0.206514

O	5.479566	0.143897	0.01955
H	5.744736	1.081216	-0.054776
O	4.093064	2.429252	-0.182496

Complex 2

1 2	x	y	z
Fe	-2.560645	0.001461	0.09347
O	-2.689437	-0.105472	-1.933534
H	-2.811834	0.722994	-2.415562
O	-4.033237	1.405908	0.230847
H	-4.915286	1.102638	-0.022773
O	-4.074212	-1.364815	0.145141
H	-4.100146	-1.907232	0.944565
O	-2.617792	-0.054905	2.120061
H	-1.8049	-0.272032	2.594781
H	-2.967194	0.754305	2.517397
H	-4.127238	-1.96722	-0.608434
H	-1.956926	-0.565951	-2.364867
H	-3.908383	2.280249	-0.159492
C	1.293852	0.035454	-0.010979
C	1.961961	1.275319	-0.039952
C	3.337187	1.293924	-0.076555
C	4.101292	0.053036	-0.084914
C	3.353487	-1.197303	-0.053466
C	1.978107	-1.195868	-0.017785
H	1.396834	2.197247	-0.033563
H	1.425033	-2.124751	0.005451
C	-0.189816	0.025947	0.02811
O	-0.843605	-1.070045	0.047666
O	-0.860831	1.106812	0.044002
O	4.065149	-2.33316	-0.060809
H	5.007893	-2.090303	-0.087675
O	5.352604	0.060943	-0.117964
O	4.034196	2.43849	-0.10622
H	4.979959	2.207162	-0.129093

Complex 2

1 6	x	y	z
Fe	-2.653373	0.080467	0.059684
O	-2.860576	-0.278428	-2.093229
H	-3.43255	0.199298	-2.706402
O	-3.96695	1.781892	0.066185
H	-4.122823	2.32693	0.84738
O	-4.110052	-1.484882	0.09951
H	-4.179263	-2.047472	0.881327
O	-2.766154	-0.246106	2.224592
H	-1.983034	-0.540419	2.705922
H	-3.322018	0.225418	2.857027
H	-4.218818	-2.042802	-0.681126
H	-2.099315	-0.583635	-2.602136

H	-4.095368	2.354136	-0.700626
C	1.36809	0.025143	-0.011692
C	2.013236	1.276952	-0.042297
C	3.388177	1.324961	-0.065473
C	4.178213	0.101573	-0.058279
C	3.45599	-1.162968	-0.026155
C	2.080554	-1.190002	-0.003698
H	1.428512	2.186317	-0.047335
H	1.546103	-2.129701	0.020175
C	-0.125797	-0.015833	0.014024
O	-0.740635	-1.1182	0.037777
O	-0.781219	1.07998	0.012466
O	4.192639	-2.284101	-0.019639
H	5.129705	-2.019597	-0.03886
O	5.430398	0.135391	-0.078833
O	4.060966	2.485051	-0.095223
H	5.011428	2.27347	-0.107212

Complex 3

1 2	x	y	z
Fe	-2.214454	-0.215865	0.009593
O	-2.686468	-0.193794	2.227783
H	-2.442843	-1.052071	2.594456
O	-3.551527	-1.977764	-0.146279
H	-4.027254	-1.97428	-0.985639
O	-3.933305	1.197018	-0.188394
H	-3.709471	1.850185	-0.862695
O	-2.203055	-0.201907	-2.293036
H	-1.393088	0.259557	-2.543309
H	-2.06304	-1.113334	-2.57648
H	-4.022583	1.70024	0.630017
H	-2.087426	0.434422	2.648937
H	-4.238138	-1.971709	0.531634
C	3.014968	-0.235283	0.025964
C	2.84391	1.155918	-0.003374
C	1.564539	1.672515	0.022997
C	0.424494	0.852952	0.077139
C	0.60276	-0.564465	0.10556
C	1.893311	-1.079445	0.080586
H	3.692062	1.830782	-0.046909
H	2.028214	-2.157706	0.102181
O	-0.487501	-1.31376	0.154487
O	-0.777453	1.368772	0.099028
O	1.367315	3.035538	-0.006382
C	4.335477	-0.841832	-0.001475
O	4.566709	-2.042669	0.0284
O	5.352322	0.045597	-0.06629
H	6.180809	-0.457543	-0.076776
H	0.405519	3.165533	0.016263

Complex 3

1 6	x	y	z
Fe	-2.220095	-0.08217	-0.076172
O	-2.521154	0.106761	2.089849
H	-2.996269	-0.608916	2.531095
O	-3.27548	-1.982821	0.092393
H	-4.120647	-2.152815	-0.34232
O	-3.923982	1.192139	-0.121677
H	-4.149043	1.722092	-0.896503
O	-2.389705	0.074262	-2.253796
H	-1.62253	0.297199	-2.795281
H	-2.933476	-0.527909	-2.776904
H	-4.194345	1.681535	0.665482
H	-1.805235	0.37064	2.680898
H	-2.737298	-2.777207	-0.020103
C	2.98835	-0.308036	0.091009
C	2.872038	1.112699	0.033776
C	1.632378	1.694215	-0.0269
C	0.45599	0.875647	-0.033547
C	0.587274	-0.578161	0.024988
C	1.880981	-1.140036	0.087394
H	3.758152	1.731628	0.038554
H	1.997754	-2.214508	0.131576
O	-0.509149	-1.256897	0.015387
O	-0.712326	1.39586	-0.090928
O	1.476325	3.03973	-0.082116
C	4.330071	-0.950368	0.157129
O	4.520262	-2.148111	0.215386
O	5.344132	-0.060645	0.145764
H	6.177846	-0.554307	0.190933
H	0.523984	3.224102	-0.117381

Complex 4

1 2	x	y	z
Fe	2.181013	-0.270248	-0.01932
O	2.315505	-0.235033	-2.034218
H	2.684045	-1.043382	-2.416899
O	3.548064	-1.768133	-0.114435
H	3.426836	-2.62075	0.323129
O	3.778019	0.95062	-0.019102
H	3.881798	1.51274	0.760627
O	2.265937	-0.192998	2.001812
H	1.616326	0.372911	2.440968
H	2.296764	-1.018734	2.503144
H	3.834378	1.524291	-0.795905
H	1.563465	0.019874	-2.584645
H	4.44711	-1.469488	0.0813
C	-3.02235	-0.144114	-0.01882
C	-2.767474	1.233327	0.001532
C	-1.465845	1.697381	0.019942

C	-0.372464	0.758842	-0.001847
C	-0.680286	-0.639923	-0.019937
C	-1.96445	-1.095113	-0.020783
H	-3.592669	1.932808	-0.006796
H	-2.1995	-2.151524	-0.014997
O	0.464851	-1.402814	-0.028735
O	0.845403	1.162183	-0.007474
O	-1.183821	3.005458	0.050037
C	-4.462472	-0.650925	-0.014334
O	-4.661515	-1.693238	0.655847
O	-5.292276	0.020984	-0.669425
H	-0.219412	3.124018	0.051751
H	0.321537	-2.351847	-0.149776

Complex 4

1 6	x	y	z
Fe	2.312257	-0.116386	0.001506
O	2.373952	-0.285649	-2.158626
H	1.761155	-0.874439	-2.617501
O	3.443451	-1.913014	-0.054172
H	3.950452	-2.232047	0.703345
O	3.905661	1.300642	0.081834
H	4.367715	1.663031	-0.68406
O	2.401127	-0.333292	2.154152
H	2.455835	0.416414	2.760301
H	1.867681	-1.006102	2.596472
H	4.486729	1.418699	0.843581
H	2.538437	0.458506	-2.751269
H	3.914612	-2.176948	-0.85495
C	-3.136382	-0.19814	-0.02649
C	-2.912725	1.185375	-0.000204
C	-1.620439	1.668718	0.029244
C	-0.499023	0.755023	0.013378
C	-0.776314	-0.654983	-0.022423
C	-2.056916	-1.125902	-0.030349
H	-3.749317	1.871399	-0.010851
H	-2.271298	-2.187023	-0.033833
O	0.369581	-1.40034	-0.042485
O	0.695259	1.205111	0.030992
O	-1.354239	2.979494	0.066437
C	-4.560717	-0.734609	-0.030962
O	-4.769676	-1.704862	0.73657
O	-5.372972	-0.156651	-0.788327
H	-0.388898	3.099361	0.076104
H	0.211529	-2.354217	-0.034771

Complex 5

1 2	x	y	z
Fe	2.183635	-0.199973	-0.019624
O	2.336026	-0.157196	-2.032329

H	2.537368	-1.018984	-2.423403
O	3.266768	-1.884203	-0.110319
H	2.872959	-2.70509	0.214511
O	3.943149	0.803727	-0.023751
H	4.149399	1.378692	0.724976
O	2.282763	-0.134375	2.000741
H	1.786845	0.561406	2.453651
H	2.112653	-0.952692	2.486696
H	4.148885	1.292788	-0.831875
H	1.639014	0.24105	-2.57044
H	4.166319	-1.832287	0.240703
C	-2.967871	-0.255138	0.015868
C	-2.801548	1.154548	0.001375
C	-1.533091	1.750775	-0.014278
C	-0.433365	0.920113	-0.013301
C	-0.55807	-0.507318	-0.004032
C	-1.868071	-1.079784	0.012937
H	-3.69356	1.770243	0.003205
H	-1.976068	-2.155806	0.022224
O	0.520187	-1.202972	-0.016271
O	0.862773	1.363337	-0.024039
O	-1.287925	3.085154	-0.029672
C	-4.395311	-0.83707	0.0352
O	-4.48265	-2.086897	0.043738
O	-5.326843	0.004945	0.040897
H	-2.106897	3.596709	-0.044251
H	0.950155	2.328751	-0.065732

Complex 5

1 6	x	y	z
Fe	2.232704	-0.208336	0.004634
O	2.415708	-0.149491	-2.191081
H	2.32357	-1.022568	-2.599803
O	3.33253	-2.076285	-0.093758
H	2.933086	-2.81019	0.398491
O	4.025463	1.082809	0.063032
H	3.945925	1.969954	-0.318735
O	2.38874	-0.216346	2.215687
H	1.954727	0.522134	2.667815
H	2.022729	-0.9985	2.654031
H	4.812455	0.729429	-0.378483
H	1.78826	0.408125	-2.673396
H	4.257767	-2.072962	0.195602
C	-3.031214	-0.245086	-0.010343
C	-2.875661	1.15739	0.016136
C	-1.608421	1.767799	0.034711
C	-0.505988	0.948158	0.024336
C	-0.620362	-0.470679	-0.002662
C	-1.912804	-1.057204	-0.017039
H	-3.770849	1.768736	0.021857

H	-2.014921	-2.133299	-0.037319
O	0.473783	-1.169539	-0.015187
O	0.806449	1.412881	0.036061
O	-1.368	3.104488	0.063993
C	-4.447737	-0.854925	-0.033498
O	-4.502738	-2.108227	-0.038481
O	-5.389584	-0.038221	-0.043403
H	-2.185814	3.618775	0.073241
H	0.847826	2.383635	0.056963

Complex 6

1 2	x	y	z
Fe	2.207982	-0.185708	0.014355
O	2.335991	-0.130306	-1.998306
H	2.516371	-0.979943	-2.424288
O	3.473310	-1.746326	-0.090598
H	3.385183	-2.469755	0.545237
O	3.853481	0.971872	-0.010964
H	4.028714	1.605035	0.698208
O	2.227914	-0.228760	2.035632
H	2.035820	0.585924	2.519657
H	1.706546	-0.926209	2.457313
H	4.001155	1.431154	-0.849473
H	1.655809	0.311822	-2.523883
H	4.393833	-1.449524	-0.039626
C	-3.089097	-0.159918	-0.010966
C	-2.902860	1.201515	-0.003332
C	-1.576458	1.774462	0.007961
C	-0.477980	0.829927	0.011894
C	-0.679847	-0.528212	0.010242
C	-1.972567	-1.040399	0.000338
H	-3.752742	1.871328	-0.007328
H	-2.158182	-2.107312	0.001360
O	0.484269	-1.295033	0.053433
O	0.803255	1.290275	0.009643
O	-1.307457	2.998793	0.005730
C	-4.506345	-0.769560	-0.023107
O	-4.558679	-2.024701	-0.035248
O	-5.460433	0.042553	-0.018996
H	0.823537	2.265952	0.002873
H	0.374136	-2.183886	-0.314063

Complex 6

1 6	x	y	z
Fe	2.357243	-0.277805	-0.017779
O	2.409075	-0.212565	-2.12818
H	2.667526	-0.98959	-2.640421
O	3.649659	-1.967357	-0.261663
H	3.43961	-2.840476	0.092927
O	3.913274	1.158328	0.315738

H	3.903101	2.033135	-0.09281
O	2.31831	-0.332616	2.09557
H	2.683198	0.391721	2.620069
H	1.652594	-0.782494	2.630968
H	4.835798	0.948647	0.509231
H	1.850829	0.341372	-2.688486
H	4.608678	-1.924804	-0.363823
C	-3.162345	-0.151595	0.03182
C	-2.960632	1.204094	-0.055533
C	-1.627023	1.752052	-0.112997
C	-0.532611	0.801062	-0.073202
C	-0.751127	-0.5549	0.0183
C	-2.05291	-1.04146	0.071663
H	-3.801224	1.884848	-0.083373
H	-2.252926	-2.103505	0.145717
O	0.390473	-1.335828	0.059122
O	0.740593	1.266179	-0.130633
O	-1.341122	2.971647	-0.193981
C	-4.583946	-0.744504	0.092207
O	-4.65011	-1.996197	0.178445
O	-5.531285	0.075267	0.049323
H	0.714852	2.242146	-0.180411
H	0.201989	-2.283967	0.076074

Complex 7

1 1	x	y	z
Fe	-2.585222	0.002071	0.023606
O	-2.66441	-0.218765	-1.999473
H	-2.773023	0.583263	-2.527239
O	-4.074495	1.402847	0.052429
H	-4.947183	1.075814	-0.203757
O	-4.097625	-1.371303	0.11652
H	-4.135308	-1.871046	0.942714
O	-2.683435	0.054509	2.051695
H	-1.870015	-0.120341	2.542639
H	-3.044274	0.883033	2.395202
H	-4.127626	-2.014021	-0.604159
H	-1.907821	-0.689957	-2.374063
H	-3.947874	2.252093	-0.389069
C	1.266567	0.05665	-0.000611
C	1.941872	1.282402	-0.074679
C	3.32939	1.292609	-0.081352
C	4.044859	0.091765	-0.015259
C	3.363899	-1.127629	0.058492
C	1.975055	-1.147389	0.065798
H	1.38229	2.20794	-0.125795
H	1.451718	-2.092275	0.123065
C	-0.209505	0.038524	0.007041
O	-0.86655	-1.058317	0.070945
O	-0.895655	1.113201	-0.048969

O	4.058619	-2.300623	0.123567
H	5.006677	-2.109528	0.108746
O	5.406942	0.053693	-0.01918
H	5.7567	0.954359	-0.071677
O	4.107048	2.417393	-0.149901
H	3.564263	3.213895	-0.188786

Complex 7

1 5	x	y	z
Fe	-2.66858	0.07612	-0.028121
O	-2.860704	-0.398449	-2.166653
H	-3.369227	0.100903	-2.817359
O	-4.015772	1.755743	-0.14823
H	-4.311685	2.274278	0.609767
O	-4.148668	-1.471089	0.072607
H	-4.254835	-1.972264	0.890976
O	-2.817068	-0.143093	2.1586
H	-2.050949	-0.523436	2.606285
H	-3.214047	0.484678	2.774669
H	-4.243657	-2.082628	-0.668442
H	-2.094693	-0.758982	-2.630408
H	-4.071285	2.334574	-0.918395
C	1.340766	0.038309	0.002267
C	1.989264	1.277772	-0.078851
C	3.376453	1.322633	-0.069395
C	4.120582	0.141892	0.02046
C	3.46803	-1.09214	0.100982
C	2.079756	-1.145431	0.092356
H	1.408291	2.188658	-0.147786
H	1.578096	-2.101709	0.155068
C	-0.143367	-0.018483	-0.006582
O	-0.759538	-1.120658	0.071563
O	-0.818743	1.069032	-0.09365
O	4.19112	-2.247556	0.188399
H	5.134046	-2.032331	0.181632
O	5.484444	0.137726	0.033326
H	5.811134	1.046356	-0.027831
O	4.125663	2.46746	-0.142821
H	3.561049	3.246982	-0.205944

Complex 8

1 1	x	y	z
Fe	2.224657	-0.244775	0.030047
O	2.225436	-0.233072	-1.989518
H	2.524832	-1.064401	-2.383362
O	3.47762	-1.817414	-0.125845
H	3.304914	-2.659613	0.315355
O	3.885102	0.884244	-0.076814
H	4.10482	1.441927	0.681677
O	2.403797	-0.151831	2.044524

H	1.811758	0.467678	2.493008
H	2.37167	-0.976384	2.548255
H	3.992367	1.418762	-0.875414
H	1.448399	0.052732	-2.488056
H	4.401496	-1.587775	0.047436
C	-3.035268	-0.191522	-0.031593
C	-2.819246	1.18979	-0.031136
C	-1.527699	1.705054	0.029068
C	-0.454947	0.821179	0.085623
C	-0.669908	-0.547541	0.086066
C	-1.949214	-1.071761	0.031514
H	-3.671774	1.856374	-0.078589
H	-2.122459	-2.140263	0.033454
O	0.495575	-1.315201	0.159742
O	0.865591	1.254152	0.150082
O	-1.196916	3.028833	0.027943
C	-4.46757	-0.748298	-0.097563
O	-4.579624	-2.00058	-0.09441
O	-5.395701	0.097908	-0.14908
H	0.364038	-2.219247	-0.157087
H	0.957952	2.187874	-0.093711
H	-1.981196	3.579328	-0.087726

Complex 8

1 5	x	y	z
Fe	2.323809	-0.246908	-0.011263
O	2.400376	-0.174355	-2.132534
H	2.636145	-0.965531	-2.633936
O	3.614654	-1.945893	-0.295739
H	3.423022	-2.80616	0.098549
O	3.925241	1.145286	0.34295
H	3.927764	2.034348	-0.033431
O	2.338629	-0.370049	2.111432
H	2.717742	0.346169	2.637086
H	1.635092	-0.777601	2.632221
H	4.847434	0.902734	0.494796
H	1.806563	0.3556	-2.679281
H	4.571321	-1.898594	-0.41637
C	-3.145613	-0.198895	0.042729
C	-2.935759	1.180507	-0.044031
C	-1.643618	1.693918	-0.094281
C	-0.560317	0.820977	-0.056326
C	-0.770122	-0.547865	0.03197
C	-2.051736	-1.070767	0.082215
H	-3.790822	1.844854	-0.07235
H	-2.221854	-2.137765	0.152875
O	0.392004	-1.308899	0.070004
O	0.748956	1.268366	-0.110117
O	-1.31767	3.018608	-0.182495
C	-4.575087	-0.760777	0.095656

O	-4.682347	-2.011503	0.175091
O	-5.510483	0.078826	0.054126
H	0.211364	-2.257912	0.084887
H	0.783786	2.23626	-0.145732
H	-2.109969	3.569177	-0.194812
